# Harnessing the immune system by targeting immune checkpoints: Providing new hope for Oncotherapy

**DOI:** 10.3389/fimmu.2022.982026

**Published:** 2022-09-08

**Authors:** Lu Yu, Minghan Sun, Qi Zhang, Qiao Zhou, Yi Wang

**Affiliations:** ^1^ Department of Pulmonary and Critical Care Medicine, Sichuan Provincial People's Hospital, University of Electronic Science and Technology of China, Chengdu, China; ^2^ Central of Reproductive Medicine, Department of Obstetrics and Gynecology, Sichuan Provincial People’s Hospital, University of Electronic Science and Technology of China, Chengdu, China; ^3^ School of Medicine, University of Electronic Science and Technology of China, Chengdu, China; ^4^ Department of Rheumatology and Immunology, Sichuan Provincial People’s Hospital, University of Electronic Science and Technology of China, Chengdu, China; ^5^ Clinical Immunology Translational Medicine Key Laboratory of Sichuan Province, Sichuan Provincial People’s Hospital, University of Electronic Science and Technology of China, Chengdu, China; ^6^ Institute of Organ Transplantation, Sichuan Provincial People’s Hospital, University of Electronic Science and Technology of China, Chengdu, China; ^7^ Department of Critical Care Medicine, Sichuan Provincial People’s Hospital, University of Electronic Science and Technology of China, Chengdu, China

**Keywords:** immune checkpoints, programmed cell death protein 1 (PD-1), programmed cell death 1 ligand 1 (PD-L1), cytotoxic T lymphocyte-associated antigen-4 (CTLA4), oncotherapy, T cell immunoglobulin and mucin-3 (Tim-3), lymphocyte activation gene 3 (Lag-3)

## Abstract

With the goal of harnessing the host’s immune system to provide long-lasting remission and cures for various cancers, the advent of immunotherapy revolutionized the cancer therapy field. Among the current immunotherapeutic strategies, immune checkpoint blockades have greatly improved the overall survival rates in certain patient populations. Of note, CTLA4 and PD-1/PD-L1 are two major non-redundant immune checkpoints implicated in promoting cancer immune evasion, and ultimately lead to relapse. Antibodies or inhibitors targeting these two c+heckpoints have achieved some encouraging clinical outcomes. Further, beyond the canonical immune checkpoints, more inhibitory checkpoints have been identified. Herein, we will summarize recent progress in immune checkpoint blockade therapies, with a specific focus on key pre-clinical and clinical results of new immune checkpoint therapies for cancer. Given the crucial roles of immune checkpoint blockade in oncotherapy, drugs targeting checkpoint molecules expressed by both cancer and immune cells are in clinical trials, which will be comprehensively summarized in this review. Taken together, investigating combinatorial therapies targeting immune checkpoints expressed by cancer cells and immune cells will greatly improve immunotherapies that enhance host elimination of tumors.

## Introduction

The concept of immunotherapy was first introduced by McFarland Burnett and Louis Thomas in 1957, who proposed that lymphocytes were the primary actors responsible for mediating immune surveillance and tumor clearance (1–3). In the ensuing decades, immunotherapy was advanced into several major areas of study, including tumor infiltrating lymphocytes (TILs), chimeric antigen receptor (CAR)-T cells, T cell receptor (TCR)-T cells, oncolytic viruses, cytokine therapy, therapeutic cancer vaccines, and immune checkpoint blocking antibodies. The goal of these treatments was to unleash the power of the host immune systems to fight back against the cancer.

With years of research and clinical trials, immune checkpoint blocking therapies have been approved by the FDA in the United States and elsewhere. Currently, administration of immunotherapies has outweighed traditional therapeutic regimes for a variety of reasons. Firstly, immunotherapy exclusively targets immune cells and tumor cells, thus leaving other cells unscathed. Secondly, the adverse effects of immunotherapy are comparatively less severe than traditional chemotherapy and radiotherapy-treatment regimens (4). However, tumor cells are highly adaptable and can become unresponsive to immunotherapies, and therefore they continuously evolve mechanisms that evade host immunity and promote tumor persistence. Furthermore, neo-antigens, a hostile tumor microenvironment, T cell exhaustion, and other factors contribute to immune checkpoint-blockade resistance (5). Nevertheless, combinatorial use of immune checkpoint blockade with a broad spectrum of chemicals and antibodies can overcome therapy resistance, and has since produced promising clinical results (6). In this review, we comprehensively summarize the recent immunotherapy literature, with a focus on immune checkpoint blockade.

## Immune checkpoints

**Figure 1:**
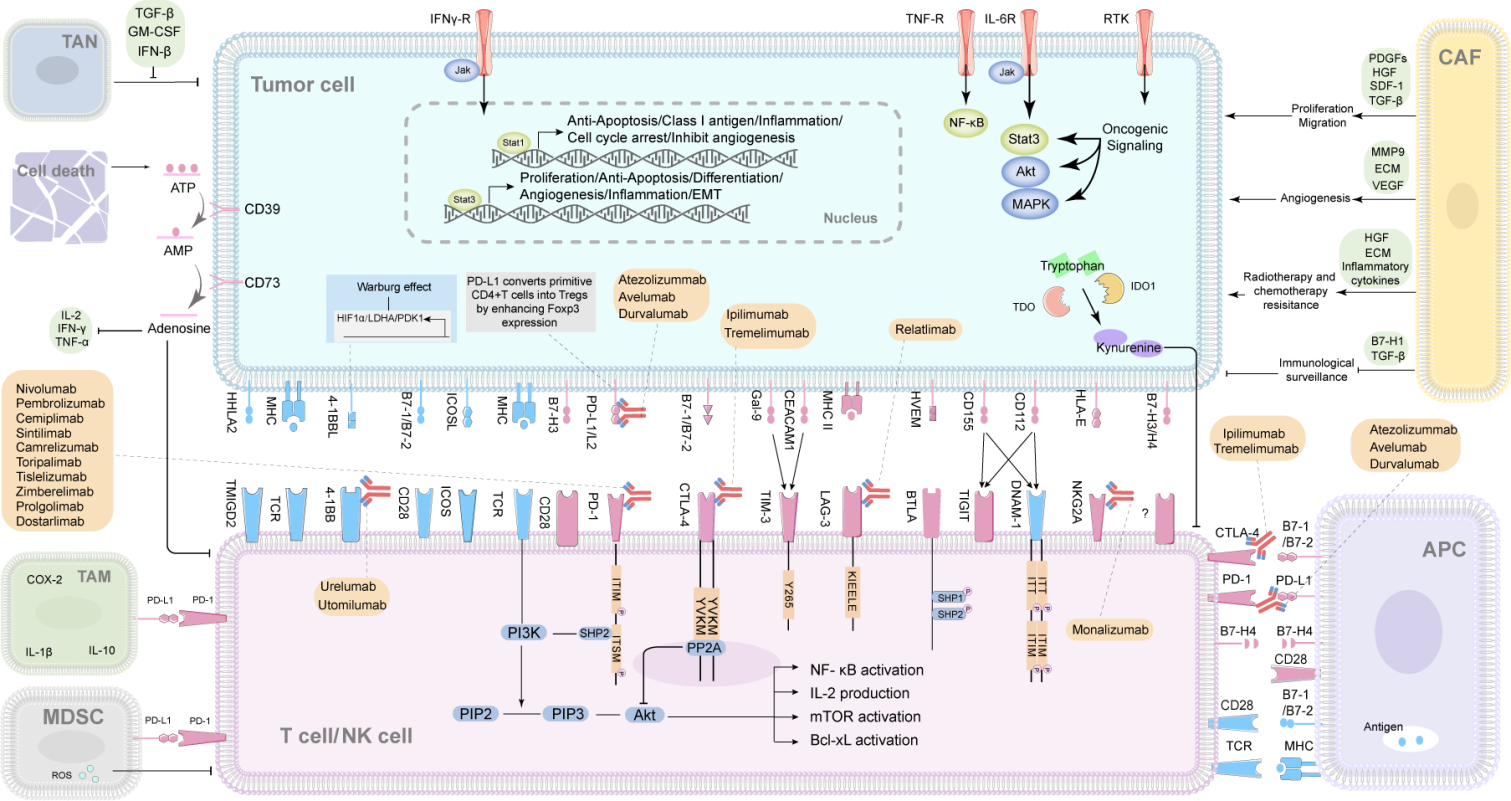
The interactions of immune checkpoints of cancers and immune cells, and the correlated immune checkpoint inhibitors (ICIs). TAN, tumor‐associated neutrophil; TAM, tumor-associated macrophage; CAF, cancer associated fibroblast; MDSC, myeloid-derived suppressor cell; APC, antigen-presenting cell; NK cell, natural killer cell; ATP, adenosine triphosphate; AMP, adenosine monophosphate; CD, clusters of differentiation; IL, interleukin; IFN-γ, interferon-γ; TNF-α, tumor necrosis factor; COX-2, cyclooxygenase-2; TGF-β, transforming growth factor-β; GM-CSF, granulocyte-macrophage colony stimulating factor; IFN-β, interferon-β; PDGFs, platelet-derived growth factors; HGF, hepatocyte growth factor; SDF-1, stromal cell-derived factor-1; MMP9, matrix metallopeptidase 9; ECM, extracellular matrix; VEGF, vascular endothelial growth factor; ROS, reactive oxygen species; TDO, tryptophan 2,3-dioxygenase; IDO1, indoleamine 2,3-dioxygenase1; HHLA2, The B7 family ligand HERV-H LTR–associating protein 2; TMIGD2, transmembrane and immunoglobulin domain containing 2; B7-, B7 family. B7-1(CD80), B7-2(CD86), B7-H1/PD-L1(CD274), B7-H2/ICOSL (CD275), B7-H3(CD276), B7-H4; 4-1BBL, 4-1BB ligand 4-1BB, CD137, a member of the TNF receptor superfamily, is an activation-induced T-cell costimulatory molecule; ICOSL, inducible costimulator ligand; ICOS, inducible costimulatory; MHC, major histocompatibility complex class; TCR, T cell receptor; PD-1, programmed cell death 1; PD-L1/L2, programmed cell death ligand-1/2; CTLA4, cytotoxic T-lymphocyte-associated protein 4; Gal-9, galectin-9; CEACAM1, Carcinoembryonic antigen elated cell adhesion molecule1; TIM-3, T cell immunoglobulin domain and mucin domain-3; LAG-3, Lymphocyte-activation gene 3; HVEM, Herpesvirus entry mediator; BTLA, B- and T-lymphocyte attenuator; TIGIT, T cell immunoreceptor with Ig and ITIM domains; DNAM-1, DNAX accessory molecule-1; HLA-E, major histocompatibility complex, class I, E; NKG2A, also called KLRC1, killer cell lectin like receptor C1; ITT, ITT-like motif; ITIM, immunoreceptor tyrosine-based inhibitory motif; ITSM, immunoreceptor tyrosine-based switch motif; PIP2, phosphatidylinositol-3,4-bisphosphate; PIP3, phosphatidylinositol-3,4,5-trisphosphate; Y265, a highly conserved tyrosine in the intracellular tail of Tim-3; KIEELE, conserved regions of Lag3 cytoplasmic domain, KIEELE motif; NF-κb, nuclear factor kappa-B; Bcl-xL, B-Cell Lymphoma-extra-large; SHP1/2, src-homology domain 2(SH2)-containing protein tyrosine phos-phatase-1/2.

As mentioned above immune checkpoints (ICPs) could overcome the limitations of conventional cancer therapy as chemotherapy and radiotherapy. Therefore, followed by the discovery of the first immune checkpoint named CTLA4, a number of ICPs, including both stimulatory and inhibitory molecules, have been identified. Systematic assessment on the correlation between the ICPs of different cancers and the treatment responses/outcomes revealed that the ICPs varied across different cancers due to the cancer heterogeneity. Meanwhile, ICPs could be potential prognostic factors and therapeutic targets in certain types of cancer (e.g. breast cancer, lung cancer and ovarian cancer) (7). Hence, we summarized the ICPs according to their pivotal role in oncotherapy. Here is the detailed information of the major ICPs (**Figure 1**).

### CTLA4

In response to foreign pathogens, T cell activation is mediated by the T cell receptor (TCR) along with the combination of co-stimulatory positive signals such as CD28 and inducible co-stimulator (ICOS), or inhibitory signals such as cytotoxic T lymphocyte-associated antigen-4 (CTLA4, also referred to as CD152) and programmed cell death protein 1 (PD-1). The final T-cell response is determined by the balance between the positive and negative signals (8). T cell activation requires 2 signals, including: 1) the engagement of the T cell receptor (TCR) with major histocompatibility molecules (MHC) on antigen presenting cells (APCs) and 2) co-stimulation *via* binding of B7(CD80/CD86) on APCs to CD28 on T cells (8). As the first immune checkpoint protein discovered, CTLA4 plays a pivotal role in immune regulation (8, 9). CD28 is predominately expressed in resting T cells, while CTLA4 is absent or scarcely expressed in resting T cells. However, following T-cell activation, CTLA4 is expressed (10). Further study revealed that the intracellular localization of CTLA4 in T cells plays an essential role in its function (11). In naive T cells, CTLA4 protein is retained in the Golgi apparatus of the cell, and therefore exerts no effect on T cell activation (12). Upon engagement of TCR with MHC, CTLA4 is translocated from the cytoplasm to the plasma membrane, where it competes with CD28 for binding with CD80/CD86 by trans-endocytosis (13). CTLA4 engagement inhibits T cell activation and proliferation to maintain immune homeostasis, and protect against aberrant immune responses to self-tissues (14). In addition, CTLA4 also induces T-cell motility and inhibits the binding of T cells with antigen presenting cells such as dendritic cells (DCs) (15). CTLA4 competes with CD28 for B7 ligands for the inhibition of effector T cells, and stimulates the transcription of Foxp3 for regulatory T cells (Tregs) (16). Furthermore, by binding to its ligands B7-1 (CD80) and B7-2 (CD86), CTLA4 also reduces T cell responses by an attenuation of the induction of memory T cells (17). CTLA deficient mice show multi-organ lymphocyte infiltration and tissue destruction, implying the critical role of CTLA4 in down-regulating T cell activation and preventing autoimmunity (18). A number of investigators have identified the cytoplasmic binding partners of CTLA4 (19). The endocytosis of CTLA4 depends largely on its adaptor clathrin, where it binds to clathrin-associated adaptor complex (adaptor protein-2, AP-2) to induce CTLA4 internalization (20). When CTLA4 is internalized, it cannot deliver a negative signal. The binding domain of CTLA4 contains a YVKM motif, and when it is phosphorylated at the tyrosine site of the YVKM motif upon T cell activation, AP-2 can disengage from the binding at the same tyrosine site of CTLA4 (20, 21). With the disengagement of AP-2, CTLA4 is activated and functions as a negative regulator of T cells.

CTLA4 also plays an important role in the regulation of regulatory T cells (Tregs) functions, including Treg suppressive function, TCR hyposignaling and the induction of anergy (22). Deficient CTLA4 expression by Tregs fails to expand effector CD4^+^ T cells, even in the presence of interleukin-10 (IL-10) (23). Treg specific CTLA4 deficiency leads to reduced expression of CD80 and CD86 on dendritic cells (DCs), lymphocyte proliferation, T-cell mediated autoimmune diseases, and immunoglobulin E (IgE) production^7 (^ 24 ^),^. CTLA4 controls both the functional-development phase and the effector phase of Tregs (25). In addition, Tregs (which constitutively express CTLA4) regulate the infiltration of CD4+ T cell into tumors *via* a CTLA4/CD80-dependent manner (26).

Previous studies revealed that CTLA4 is constitutively expressed on tumor cells, and, therefore, CTLA4 blockade might stimulate tumor cell apoptosis and lead to the regression of tumors (27). Because CTLA4-inhibited immune activation relies primarily on the stimulation and activation of Tregs, the therapeutic effect of CTLA4 blockade principally acts *via* the regulation of Tregs (28). Administration of neutralizing monoclonal antibodies against CTLA4 are able to selectively deplete FOXP3^+^ Treg cells, and thereby promote tumor immunity by activating effector CD8^+^ T cells (29). Clinically, treatment with anti-CTLA4 monoclonal antibodies (mAbs) does not deplete Foxp3^+^ Tregs, but does increase CD4^+^ and CD8^+^ T cell infiltration into tumors (30).

### PD-1 and PD-L1

Programmed cell death protein 1 (PD-1) was named based on its initial identification as a receptor inducing cell death in an activated T cell hybridoma (31), and is now recognized as a dominant negative regulator of T cells similarly to CTLA4 (32). Like CTLA4, PD-1 is absent from naïve and memory T cells, but is expressed upon TCR engagement (33). In contrast to CTLA4, PD-1 is expressed on the surface of activated T cells, and contains a conventional immunoreceptor tyrosine inhibitory motif (ITIM) and immunoreceptor tyrosine switch motif (ITSM). PD-1 engagement leads to the activation of the inhibitory phosphatase SHP-2, thus resulting in the inhibition of TCR mediated function, increased T cell migration, and immune evasion within the tumor microenvironment (TME) (34). Unlike the ligand of CTLA4, one of PD-1’s ligands known as programmed death- ligand 1 (PD-L1), is broadly overexpressed on tumor cells and infiltrating leukocytes (35); this allows for the induction of PD-1 mediated T cell exhaustion by tumor cells (36). However, this phenotype can be reversed *via* blockade of either PD-1 or PD-L1, resulting in elevated antitumor cytotoxic T cell responses and tumor regression (35). Because CTLA-4 inhibits T cells at the early stages in the lymph nodes, the CTLA4 deficient mice show severe lymphoproliferative disease, lethal lymphocytic infiltration in multiple organs and tissue destructions (37). However, because PD-1 regulates in the immune response in the peripheral tissues, the phenotypes of PD-1 and PD-L1 deficient mice are comparatively milder, as evidenced by delayed onset of inflammation and lack of severe organ destruction. These differences highlight the importance of the PD-1 pathway, specifically, in tumor immunotherapy (38, 39).

It is widely acknowledged that CTLA4 regulates T-cell proliferation in lymph nodes (40). Therefore, CTLA4 antibodies (ipilimumab and tremelimumab) can induce clinically unrestrained T-cell activation (41). And the major immune-related adverse events (irAEs) of CTLA4 blockade have been reported to be diarrhea, dermatitis, hepatitis, and endocrinopathies. However, PD-1 suppresses T cells primarily in peripheral tissues (40), and, hence, blockade of PD-1 or PD-L1 exerts less frequent and mild autoimmune adverse effects compared with CTLA4 mAb therapies (35). However, the therapeutic outcomes of PD-1 blockade on solid tumors have remained relatively poor, mainly due to the hostile TME, and the presence of immunosuppressive Tregs (42). PD-L1 has been implicated in the development and function of Tregs by enhancing the expression of Foxp3. It also converts naive CD4^+^ T cells to Tregs, and stimulates the immunosuppressive function of Tregs (43). The expansion of Tregs could be regulated by PD1 and PD-L1 *via* Notch signaling pathway (44). Additionally, in myeloid tumor cells (K562) or T cells, the PD-L1 expression was able to convert Th1 cells into Tregs (45). Furthermore, PD-1 blockade stimulates the proliferation of PD-1^+^ effector Treg cells, inhibiting the antitumor immunity (46, 47). Therefore, the efficacy of PD-1 blockade therapy may be predicted by the levels of PD-1 expression on effector T cells (Teff) and Tregs (48), and should be used as a biomarker to predict patient responsiveness.

### Tim-3

T cell immunoglobulin and mucin-3 (Tim-3; also known as HAVCR2) is broadly expressed on activated T cells, FoxP3^+^ Treg cells, NK cells and monocytes (49). Tim-3, similarly to PD-1 and CTLA4, suppresses the immune response upon interaction with its ligand galectin-9 (49). The main functions of Tim-3 include promoting CD8+ T cell exhaustion, and inducing the expansion of myeloid-derived suppressor cells (MDSCs) and Tregs. Co-expressed with carcinoembryonic antigen cell adhesion molecule 1 (CEACAM1), the maturation and cell surface expression of Tim-3 is regulated by CEACAM1 by forming a heterodimer to mediate immune tolerance and T cell exhaustion (50). Blockade of Tim-3 accelerates the pathogenesis of autoimmune diabetes in nonobese diabetic (NOD) mice, and inhibits transplantation tolerance induced by co-stimulation blockade (51), highlighting Tim-3’s immunoregulatory role. As a T helper type 1 (Th1)–specific cell surface marker, Tim-3 binds with its ligand galectin-9, causes the hyperproliferation of Th1 cells and the release of Th1 cytokine, and thereafter induces peripheral tolerance (52, 53). Activation of the Tim-3/galectin-9 pathway is also known to increase the frequency of CD11b^+^Ly-6G^+^ myeloid cells, inhibits immune responses (54). Clinically, Tim-3 promotes T-cell exhaustion and is associated with the poor prognosis in hepatocellular carcinoma (HCC) patients (55). Tim-3 blockade also increases the pathological severity of the Th1-dependent autoimmune disease of experimental autoimmune encephalomyelitis (EAE) in mice (56).

Regarding Tregs, Tim-3^+^ Treg is highly effective at inhibiting T-cell proliferation (57, 58), and the blockade of Tim-3 induces a reduction in the frequency of CD4^+^CD25^+^Foxp3^+^ Tregs (59). Experiments using murine tumor xenograft models suggest that Tim-3 is expressed on PD-1 expressing CD8^+^ TILs. Functionally, Tim-3+PD-1+ TILs are severely exhausted, as evidenced by a failure to secrete interferon-γ (IFN-γ), tumor necrosis factor-α (TNF-α), and interleukin-2 (IL-2) (60). Therefore, compared with the inhibition of CTLA4 pathway or PD-1/PD-L1 pathway alone, combinatorial Tim-3 and PD-1 blockade has been demonstrated to be more effective (60). For example, combinatorial therapy reverses tumor-induced T-cell exhaustion and dysfunction in patients with colorectal cancer by increasing the proliferation of tumor antigen-specific CD8^+^ T cells and decreasing immunosuppressive Treg populations (61). In patients with advanced melanoma, increased expression of Tim-3 and PD-1 correlates with tumor antigen-specific CD8+ T cell dysfunction (62). A recent study using triple therapy (PD-1 blockade, Tim-3 blockade and anchored granulocyte- macrophage colony- stimulating factor (GM-CSF) vaccination) demonstrated tumor regression levels higher than 50% (63). This was associated with reduced apoptosis of CD8^+^ TILs, decreased production of tumor-promoting cytokines, and improved cytotoxicity in bladder cancer (64) and prostate cancer (65). In another study, combinatorial Tim-3/Lag-3/PD-1 blockade significantly improved antitumor immunity in gastric cancer cell-T cell coculture models, suggesting the therapeutic potential of combinatorial therapy in gastric cancer patients (66). Tim-3 could also be used as a biomarker for cancer therapy responsiveness. In medullary thyroid cancer, a cohort study of 200 patients revealed that Tim-3, CTLA4 and PD-1/PD-L1 are promising biomarkers for tumor recurrence (67). In head and neck cancer, increased PD-1^+^ and Tim-3^+^ CD8^+^ TILs were inversely correlated with clinical outcome of cetuximab therapy (68). Ultimately, Tim-3 blockade has been proven in pre-clinical studies to be beneficial in improving current immune-checkpoint blockade therapies when applied in combination. These promising results provide precedence for assessing the use of Tim-3 blockade in clinical trials so as to improve clinical outcomes of cancer patients.

### Lymphocyte activation gene 3

Discovered in 1990, lymphocyte activation gene-3 (Lag-3; also referred to as CD223), is an immune checkpoint molecule expressed by activated T cells, Tregs, NK cells and B cells (69–72). Lag-3 is structurally homologous to the CD4 co-receptor and competes with CD4 for its binding with MHC class II (73). Inhibition of Lag-3 results in the expansion of T cells, B cells, macrophages, granulocytes, and DCs (74), and increases activation of CD8^+^ T cells (75). With respect to Lag-3’s regulatory functions, a study by Workman et al. revealed an inability of Vβ7/8^+^ (T cells bearing Vβ7 and Vβ8 (Vβ7/8) T cell receptors) Lag-3-deficient T cells to expand, even when stimulated with staphylococcal enterotoxin B. Expansion of Lag-3 deficient T cells was subsequently restored by the constitutive expression of Lag-3 (76). Lag-3 is differentially expressed on induced Tregs and is required for Treg function. Upon activation, natural CD4^+^CD25^+^ Tregs express Lag-3 to suppress effector cells, whereas CD4^+^CD25^+^ Tregs from Lag-3 knockout mice exhibit reduced regulatory activity (77). Lag-3 is also a biomarker for active CD4^+^CD25^high^Foxp3^+^ Tregs (78). CD49b and Lag-3 are stably and selectively co-expressed on CD4^+^ type 1 Tregs (Tr1), which induce and maintain immune tolerance (79). Moreover, Lag-3 is expressed on CD11c^l^°^w^/B220^+^/PDCA-1^+^ plasmacytoid DCs, and regulates their homeostasis (80). Lag-3 binds with MHC II expressed within plasma membrane lipid rafts of immature human DCs, and promotes their maturation and activation (81). Due to the pivotal role of Lag-3 in immune regulation, several diseases including autoimmune diseases, cancer, chronic viral infection, and parasitic infection are correlated with aberrant Lag-3 expression (82–84). Given the immunoregulatory role of Lag-3, the combinatory therapies targeting both Lag-3 and PD-1 have gained some encouraging clinical outcomes (85–87). Lag-3 and PD-1 contribute to the rapid trafficking of the immunological synapse upon T-cell activation, and lead to a synergistic inhibitory effect on T-cell signaling (88). Dual blockade of Lag-3 and PD-1 resulted in the clearance of multiple established tumors, which were resistant to PD-1 blockade alone (89). Another study in renal cell carcinoma revealed that the inhibition of PD-1 resulted in a significant upregulation of Lag-3, and the blockade of both immune checkpoint proteins leads to increased IFN-γ secretion, emphasizing the significance and the potential of combination therapy (90). Therefore, to overcome the resistance of PD-1 blockade in resistant cancers, the synergistic blockade of Lag-3 and PD-1 may provide a promising therapy.

### TIGIT

Discovered by genome-wide association studies (GWAS), the co-stimulatory molecule CD226 together with T cell immunoreceptor with immunoglobulin and ITIM domain (TIGIT) forms a striking pathway similarly to the CD28-CTLA4 pathway, and is correlated with multiple autoimmune diseases (91, 92). TIGIT is expressed by the activated T cells, Tregs, and NK cells (93). The ligand for TIGIT is poliovirus receptor (PVR) CD155, which also binds to CD226. Hence, in the TIGIT-CD226 axis, TIGIT can bind and disrupt CD226 homodimerization, and therefore directly inhibits T-cell activation, proliferation, and effector function by competing with CD226 for binding to CD155 (94, 95). TIGIT has been shown to suppress antitumor immunity by increasing the frequency of Tregs that express the co-inhibitory receptor Tim-3 and that induce CD8^+^ T-cell dysfunction (96). TIGIT^+^ Tregs selectively suppress the responses of proinflammatory Th1 and Th17 cells *via* the upregulation of fibrinogen-like protein 2 and the secretion of IL-10 (97). TIGIT represses IFN-γ secretion, promotes the nuclear translocation of forkhead box O1 (FoxO1), inhibits protein kinase B (PKB, also called Akt) function, and restores suppressor function of Tregs (98). TIGIT also plays an important role in regulating NK cell responses. In mouse models, the blockade of TIGITs interaction with its ligand PVR leads to increased NK-cell mediated cytotoxicity (99). In tumor models, TIGIT exerts its regulatory function and inhibits NK-mediated cytotoxicity on tumor cells to promote immune evasion (100). Antibody blockade of TIGIT could inhibit NK cell exhaustion and promote NK cell dependent tumor immunity (101). Clinically, dual blockade of TIGIT and PD-1 could increase the frequency of CD8^+^ TILs and tumor antigen specific CD8^+^ T cells in established melanoma patients (102). Dual PD-1 and TIGIT blockade treatment in glioblastoma multiforme (GBM) patients showed decreased effector T cell function, increased Tregs, and increased tumor infiltrating dendritic cells (TIDCs) (103). In B-cell non-Hodgkin’s lymphoma patients, the response rates (RRs) of PD-1 blockade are comparatively low, and these patients display increased CD8^+^ and CD4^+^ T effector memory cells expressing TIGIT and PD-1 with limited IFN-γ, TNF-α, and IL-2 production (104). In gastric cancer patients, TIGIT and PD-1 were found to be upregulated on infiltrating CD8^+^ T cells in tumor tissues, suggesting that TIGIT may serve as an emerging biomarker (105). In follicular lymphoma, increased numbers of TIGIT^+^ T cells are associated with poor survival, therefore TIGIT may serve as a predictive marker for therapeutic clinical outcomes (106).

### BTLA

The B and T lymphocyte attenuator (BTLA) is an immunoglobulin superfamily member, which downregulates T-cell activation (69). BTLA contains a glycoprotein with two immunoreceptor tyrosine-based inhibitory motifs. The ligand for BTLA is B7x, a peripheral homolog of B7. BTLA is not expressed in naïve T cells, but it is expressed on Th1 cells upon activation (107). After crosslinking BTLA with antigen receptors, BTLA attenuates IL-2 secretion, and therefore it functions as an inhibitory receptor similar to CTLA4 and PD-1 (108). Blockade of BTLA in T-cells increased T-cell proliferation, and BTLA deficient mice display increased incidence and severity of autoimmune diseases, such as EAE. A recent report suggests that PD-1 and BTLA can suppress T cell signaling *via* SHP-1 and SHP-2 (109). BTLA also functions as a negative regulator of B cell receptor (BCR) signaling by binding with its ligand herpesvirus entry mediator (HVEM). Engagement of BTLA4 leads to recruitment of the tyrosine phosphatase Src homology 2 domain containing phosphatase 1, and reduces the activation of BCR-signaling pathways (108). Furthermore, BTLA4 engagement inhibits the activation of B cells by targeting Syk and B cell linker proteins (110).

BTLA is also expressed on DCs and macrophages (111, 112). BTLA plays a regulatory role in peripheral tolerance, evidenced by the ability of BTLA^+^ DCs to induce CD8^+^ T cell tolerance that could alleviate the severity of type 1 diabetes (113). Regarding Tregs, BTLA governs the cell differentiation and activation of Tregs (114). BTLA deficient mice have fewer Tregs and develop autoimmune diseases such as EAE. Interestingly, adoptive transfer of myelin oligodendrocyte glycoprotein fused reovirus protein σ1-B220^+^CD5^+^ Bregs, which express elevated BTLA levels, protects against EAE development, suggesting that BTLA plays a critical role in the activation of Tregs and Bregs (115). Therapeutically, several studies have explored the application of BTLA4 blockade in improving clinical outcomes in patients with colorectal cancer, epithelial ovarian cancer, lung cancer, gastric cancer, and other cancers (116–119). BTLA blockade inhibits IL-6 and IL-10 secretion, induces CD19^hi^ B cells through AKT and STAT3 signaling pathways, and significantly improves the therapeutic outcomes in epithelial ovarian carcinoma patients (117). Another study revealed that up-regulation of BTLA occurs independently of functional exhaustion, which is induced by high antigen load. Combinatorial blockade of PD-1, Tim-3 and BTLA could enhance the expansion, proliferation, and cytokine production of CD8^+^ T cells (120). Taken together, BTLA should be further explored to enhance the clinical responsiveness to immune checkpoint blockade therapies.

### Other immune checkpoints

In addition to the immune checkpoints mentioned above, other immune checkpoint molecules have been identified including B7 family molecules (B7-H3, B7-H4 and HHLA2), indoleamine 2,3-dioxygenase 1 (IDO-1), V-domain Ig suppressor of T cell activation (VISTA), and inducible T cell co-stimulator (ICOS). B7-H3, also known as CD276, is a newly discovered immune checkpoint protein, and plays a crucial role in the adaptive immune response in human cancers (121, 122). As a transmembrane glycoprotein, B7-H3 contains single variable and several constant immunoglobulin domains. It binds with its receptor CD28 which is universally expressed on T cells, B cells, monocytes, NK cells, and APCs including DCs and myeloid-derived suppressive macrophages (123, 124). Moreover, it is also constitutively expressed on the surface of tumor cells fibroblasts, tumor endothelial cells, osteoblasts and amniotic fluid stem cells (125). Upon interaction with CD28, B7-H3 ultimately leads to immune evasion by inhibiting the activation of T cells. B7-H3 deficient mice have increased frequencies of differentiated Th1 cells associated with severe inflammation in the respiratory system and the development of autoimmune encephalomyelitis earlier than wild type littermates (126). However, B7-H3 also possesses a co-stimulatory role by inducing IFN-γ production, increasing T cell proliferation (121), and stimulating the differentiation of CD8^+^ T cells that drive antitumor immunity (127). CD4^+^CD25^+^Tregs inhibit DC activation *via* up-regulation of inhibitory B7-H3 on DCs. B7-H3 engagement also results in decreased MHC-peptide complexes and impairs T cell activation (128). The expression of B7-H3 and B7-H4 has been reported to be correlated with metastasis by stimulating the expression of immunosuppressive IL-10 and TGF-β1 (129). Studies in tumor metabolism revealed that B7-H3 increases the expression levels of hypoxia-inducible factor 1 α (HIF1α), lactate dehydrogenase A (LDHA), and pyruvate dehydrogenase kinase 1 (PDK1), and therefore stimulates the Warburg effect by increasing glucose uptake and lactate production (130). These studies provide evidence that B7-H3 can regulate aspects of both cancer persistence and immune regulation, and should be considered as a potential target in the next stage of immune checkpoint blockade therapies for cancer.

B7-H4, also referred to as B7x and B7-S1, is a highly conserved immunoglobulin superfamily and belongs to the B7 family (131). It is ubiquitously expressed by tumor tissue and immune cells, including T cells, APCs, DCs, peritoneal macrophages and B cells (131, 132). B7-H4 negatively regulates T cell activation by decreasing T cell proliferation, inhibiting the production of IL-2 and inducing cell cycle arrest of T cells (133, 134). B7-H4 regulates pro-inflammatory T-cell responses by inhibiting CD4^+^ T-cell proliferation and differentiation to both Th1 and Th17 cells, inducing the production of IL-10, and increasing Treg activation (135). Deficiency of B7-H4 leads to elevated Th1 and Th17 responses (136). The expression of B7-H4 was shown to be up-regulated in various cancers, including breast, pancreatic, ovarian, lung, renal cell, and endometrial cancers (137–140). The expression levels of B7-H4 have also been recognized to constitute a prognostic indicator associated with poor overall survival in pancreatic cancer patients (137).

HHLA2 (HERV-H LTR-associating 2; also referred to as B7H5 and B7H7) belongs to the B7 family and is expressed by macrophages and DCs. As a novel co-stimulatory molecule, HHLA2 binds with CD28H (also known as Transmembrane and Immunoglobulin Domain Containing 2 (TMIGD2)), stimulates T cell proliferation and increases cytokine production *via* induction of the Akt signaling pathway (141–143). When combined with TCR and CD28 stimulation, it plays a co-inhibitory role comparable to PD-L1 (144). In T cells and NK cells, it also binds with KIR3DL3 (killer cell immunoglobulin-like receptor, three immunoglobulin domains and long cytoplasmic tail 3) to mediate tumor immune evasion independently from PD-L1 (145). HHLA2 inhibits T-cell proliferation and cytokine production including IFN-γ, TNF-α, IL-5, IL-10, IL-13, IL-17A, and IL-22 (146). Therefore, it has been reported to be a novel prognostic predictor, similar to PD-1, in tumor immunotherapy (147, 148).

V-domain Ig-containing Suppressor of T cell Activation (VISTA; also referred to as PD-1H, DD1α, c10orf54, Gi24, Dies1 and SISP1) is a transmembrane protein containing a single N-terminal immunoglobulin (Ig) V domain (149). Phylogenetically, VISTA is similar to PD-1, CD28, and CTLA4 (150). It is the most conserved molecule among the B7 family members, sharing similar domains with CD28 and CTLA4 within its cytoplasmic tail (149). Studies on VISTAs Src homology 2 (SH2) binding motif suggest that it functions as both a ligand and receptor in controlling immune responses (149, 151). VISTA is mainly expressed on cells of the hematopoietic lineage, including macrophages, DCs, monocytes, and circulating neutrophils (152). Albeit absent in B cells, it is unanimously expressed in naïve T-cells, CD4^+^ memory T- cells and Tregs (150). Weak expression of VISTA is also observed in CD8^+^ T cells and NK cells (149). VISTA deficiency results in enhanced activation of T-cells with increased production of IFN-γ, TNF-α and IL-17, and contributes to the onset of overt autoimmunity (151, 153). Within the TME, VISTA plays a crucial role with increased expression observed on myeloid DCs, myeloid-derived suppressor cells (MDSCs) and Tregs (154). Blockade of VISTA decreases the number of MDSCs, increases activated DCs, and reduces the frequency of tumor specific Tregs (155).

The immune checkpoint molecules indoleamine 2,3-dioxygenase-1 (IDO-1) and tryptophan dioxygenase (TDO) belong to the tryptophan catabolic enzyme family. They catalyze the degradation of tryptophan (Trp) into kynurenine (Kyn) (156, 157). IDO-1 plays an immunosuppressive role in various cancers (158) by suppressing CD8^+^ T effector (Teff) cells and NK cells (159, 160). Further, it stimulates the proliferation of Tregs and MDSCs (161), decreases the ratio of Th17 cells versus Tregs, inhibits Th17 related cytokine production, and decreases the number of Th1 and Th22 cells (162, 163). Moreover, IDO-1 regulates the recruitment, polarization and phagocytosis capabilities of macrophages (164, 165). TDO has also been reported to be an immunosuppressive molecule capable of reducing CD8^+^ T cell viability, stimulating the secretion of cytokines such as IFN-γ, TNF-α, IL-10 and IL-17 by TDO reactive CD4^+^ T-cells, and contributes to the tumor metastasis (166–168).

Inducible Co-Stimulator (ICOS), a homodimeric protein expressed on activated T cells, is an inducible T-cell co-stimulator (169). Specifically, it shares common signaling mechanisms with CD28, and increases T cell responses by stimulating T-cell proliferation and cytokine secretion (169). By binding with its ligand (B7-H2, B7RP-1), ICOS stimulates the response of T effector cells and T cell-dependent B cells (170). Further, this interaction promotes IL-10 secretion by effector T cells even in the presence of mature DCs (171). ICOS blockade attenuated T cell proliferation and inhibited Th effector responses by decreasing Th2 cytokine secretion (172). Inhibition of ICOS attenuates cytokine productions as IL-2, IL-4, IL-5, and IFN-γ, and decreases the Th2 mediated mucosal inflammation (173). Deficiency in ICOS fails to form T follicular helper (Tfh) cells. Therefore, inhibition of ICOS decreases the immunosuppressive function of Tregs and decreases the expression of Tfh markers of the lymphoid tumor cells (174, 175). Beyond the canonical immune checkpoints of CTLA4 and PD-1/PD-L1, other immune checkpoints may, therefore, also be crucial in reducing immune responses in numerous cancers. Combination therapy with classical inhibition of CTLA4 and PD-1/PD-L1 may, thus, result in a durable clinical response in cancer therapy.

## Drugs targeting the immune checkpoints

Tremendous progress has been achieved in cancer immunotherapy over the past few decades. To date, the most widely applied immunotherapeutic drugs are antibodies that block immune inhibitory receptors such as CTLA4, PD-1, and PD-L1 (**Supplementary Table 1**). Although the FDA has approved these drugs to treat various cancer types, a large number of immune checkpoint inhibitors (ICIs) targeting other inhibitory receptors are in clinical development. In this section, we will summarize these drugs in cancer immunotherapy.

### CTLA4 inhibitors

Ipilimumab was the first FDA approved CTLA4 inhibitor to hit the market (176). In humans, a phase III clinical trial in 2010 (MDX010-20, NCT00094653) in patients with unresectable or metastatic melanoma evaluated the efficacy and safety of ipilimumab (3 mg/kg) and found it alone extended the median overall survival (OS) of patients by more than three months compared with the control group, showing that a single ICI can have potent antitumor effects (177). In 2011, ipilimumab was approved by the FDA for the treatment of late-stage (metastatic) melanoma patients (176), and 3 mg/kg administered once every 3 weeks (Q3W) for four cycles was adopted as the standard of care based on the results of several clinical trials (177–179). Besides melanoma, clinical trials using single-dose, 3 mg/kg ipilimumab have been conducted in patients with ovarian cancer (180, 181) and prostate cancer (182), but the its effect was not as effective in these cancer types compared to the success rates for melanoma patients. Thus, use of ipilimumab in combination with other treatment modalities, such as additional immunotherapies, chemotherapies and radiotherapies, are being tested in the clinic to improve success rates in multiple cancers. For example, 10 mg/kg ipilimumab plus dacarbazine (a chemotherapeutic agent) prolonged the median OS from nine months to 11 months in patients with previously untreated metastatic melanoma compared with dacarbazine plus placebo (183).

Another CTLA4 inhibitor, tremelimumab, is a fully human mAb containing an IgG2 Fc domain in order to minimize Fc domain effector functions such as complement fixation and antibody-dependent cytotoxicity (184, 185). Tremelimumab alone has displayed potential antitumor effects in advanced melanoma patients in phase I, I/II and II trials (186–188). However, in a phase III trial in patients with unresectable stage IIIc–IV melanoma who had not received prior systemic treatment, tremelimumab was found to not be superior to standard of care chemotherapy regimens (median OS 11.8 months vs 10.7 months, hazard ratio (HR) 0.96) (189). These disappointing results led to early termination of the study and has limited available data of tremelimumab in melanoma patients. Besides melanoma, tremelimumab monotherapy has been studied in several other solid tumors, including metastatic esophageal and gastric adenocarcinoma, locally advanced or metastatic non-small cell lung cancer (NSCLC), treatment-refractory colorectal cancer (CRC), etc, but no satisfactory results have been obtained (190–193).

### PD-1 inhibitors

Currently there are three anti-PD-1 and three anti-PD-L1 antibodies approved by the FDA for treatment of more than 10 cancer indications. The first PD-1 inhibitor to enter the clinic was nivolumab in 2006, and the first to receive FDA approval was pembrolizumab in 2014 for the treatment of advanced melanoma patients (194). Both of these PD-1 inhibitors contain human IgG4 Fc regions containing mutations in the stabilizing hinge region (195). Nivolumab was tested in 2010 in a phase I trial in 39 patients with advanced solid tumors that were treatment-refractory (196). These tumor types included metastatic melanoma, prostate cancer, NSCLC, renal cell carcinoma (RCC) and CRC. Different doses (0.3, 1, 3, 10 mg/kg) of nivolumab were used and antitumor activity was found in 1 CRC (complete response (CR), 3 mg/kg), 1 melanoma (partial response (PR), 10 mg/kg) and 1 RCC patient (PR, 10 mg/kg). The drug was well-tolerated and common AEs observed were reduced lymphocyte numbers, fatigue, and musculoskeletal symptoms (196). In a subsequent study, a multidose (ranging from 0.1 to 10 mg/kg) clinical trial with expanded patient numbers (N=296) was conducted in the same tumor types (197). CR or PR was observed in melanoma (28%), RCC (27%) and NSCLC (18%) patients, and phase III trials were then initiated for these indications (198–201). Considering the promising results from these trials, the FDA approved nivolumab for the treatment of refractory melanoma in 2014 and NSCLC in the following year (202). Since then, nivolumab has gained FDA approval for the treatment of multiple solid tumor types (**Supplementary Table 1**). Interestingly, in classical Hodgkin’s lymphoma (cHL), the surface of tumor cells also overexpresses PD-L1/PD-L2 due to genetic alterations on chromosome 9p24.1 (203, 204). In a phase I study, nivolumab showed beneficial responses in 87% (20/23) of relapsed/refractory cHL patients (205). In a phase II study, the overall objective response rate (ORR) was 69% and median PFS was 14.7 months (206). Thus nivolumab was also approved for treating cHL patients in 2016 as the first PD-1 inhibitor (207).

Pembrolizumab was produced by grafting the variable region sequences of a mouse mAb onto a homologous human IgG4-κ isotype framework containing a stabilizing S228P Fc mutation to reduce the immunogenicity of the mouse variable region (208). In a Phase I trial of patients with advanced ipilimumab-refractory melanoma, 26% of patients in the pembrolizumab group showed either CR or PR (209). Later in a Phase III trial in patients with advanced melanoma, the RR was significantly higher in pembrolizumab 10 mg/kg Q2W (33.7%) and Q3W (32.9%), compared with ipilimumab (11.9%) (210). In NSCLC patients (untreated, advanced), a Phase III trial showed the median PFS was 10.3 months in pembrolizumab, compared to 6.0 months in chemotherapy groups and the RR was 44.8% and 27.8%, respectively (211). Pembrolizumab was well tolerated, and the most common AEs reported were fatigue, pruritus, and rash. Similar to nivolumab, pembrolizumab is approved for treatment of patients with multiple cancer types (202).

Cemiplimab is another human IgG4 antibody that is approved for treating cutaneous squamous cell carcinoma (cSCC), basal cell carcinoma and certain types of NSCLC (212, 213). In a meta-analysis comparing the efficacy of cemiplimab and pembrolizumab in advanced cSCC patients, cemiplimab treatment was associated with better OS (HRs ranging from 0.21–0.52) and PFS (HRs ranging from 0.49–0.55) (214). However, this conclusion should be interpreted with caution as its results were from indirect comparison without evidence from head-to-head clinical trials. The overall number of comparator studies included in this meta-analysis was low, and some studies enrolled small numbers of patients. While data from nivolumab or pembrolizumab trials treating locally advanced or metastatic vulvar SCC has been scarce, a phase III trial (NCT03257267) investigated cemiplimab in patients with recurrent/metastatic (RM) cervical cancer, who were resistant to first-line platinum-based therapy. Results from the overall population highlighted a 31% reduction in the risk of death as well as a 25% reduction in disease progression and an ORR of 16% (n = 50) [6% (n = 19) in the chemotherapy-treated cohort]. Further, the median OS in the cemiplimab group was 12 months compared to 8.8 months with chemotherapy, and the drug was well tolerated (215). According to these data, cemiplimab was granted priority review by the FDA for patients with RM cervical cancer who have experienced disease progression while on or after chemotherapy treatment regimes.

Besides the anti-PD-1 mAbs discussed here, a number of other anti-PD-1 mAbs have been approved by China or the European Union for treatment of different cancers, such as sintilimab, camrelizumab, toripalimab, tislelizumab, zimberelimab, prolgolimab, or dostarlimab; all of which have shown promising therapeutic effects for various cancer related indications (216).

In addition to PD-1 blocking agents, antibodies targeting PD-L1 also have potent antitumor qualities. Currently, three anti-PD-L1 antibodies, atezolizumab, avelumab, and durvalumab, are approved by the FDA to treat several cancers (217). Atezolizumab is a human IgG1 that has an N297A mutation to eliminate the unwanted fragment crystallizable (Fc)-mediated functions such as antibody-dependent cytotoxicity (ADCC) by aglycosylation (218). Durvalumab also contains a modified Fc region to prevent depletion of PD-L1 expressing T cells (217). In contrast, avelumab owns a native Fc region which can bind to Fc-γ receptors on NK cells to initiate ADCC (217). Their approved indications, time and dosage are summarised in **Supplementary Table 1**.

### Non- CTLA4 and PD-1 ICIs

Despite the success of CTLA4 or PD-1/PD-L1 inhibition, a majority of patients develop drug resistance. Thus, the identification of novel and non-redundant pathways of immune inhibition is important for advancing therapies that promote anti-tumor immunity. In a phase III clinical trial of anti-Lag-3 (relatlimab, 160 mg Q4W) combined with nivolumab (480 mg Q4W) for melanoma patients, patients receiving dual Lag-3/PD-1 blockade showed an increased PFS than those receiving nivolumab alone (median PFS 10.1 months vs 4.63 months, and PFS rates 47.7% vs 36.0% at 12 months). The combined regimen was well tolerated and total AEs were not significantly increased: 18.9% vs 9.7% grade 3/4 TRAEs and 14.6% vs 6.7% treatment discontinuations (219). Thus, this fixed-dose combination trial laid the foundation for dual inhibition apart from anti-CTLA4/anti-PD-1 pathways for clinical use and was recently FDA approved for patients with unresectable or metastatic melanoma (220).

Monalizumab is a humanized anti-NKG2A antibody that has the ability to enhance the anti-tumor activity of NK cells (221). In A20 tumor-bearing mice, combined treatment with anti-NKG2A and anti-PD-L1 mAbs rescued 75% of the mice from death compared to 40% in the anti-PD-L1 alone group (221). Although monalizumab alone was tested in patients with RM squamous cell carcinoma of the head and neck (SCCHN) in a phase II trial (222), the study did not meet its primary endpoint (no OR was observed) and it was terminated at the interim as ineffective (222). The safety profile of monalizumab was favorable, and it is now being tested in combination with durvalumab in a biomarker-based study in RM SCCHN patients. Cetuximab, an anti-EGFR mAb, promoted ADCC by binding to CD16/FcγRIII (223), and the ADCC induced by cetuximab was enhanced by monalizumab through NK cell stimulation, providing greater anti-tumor activity than cetuximab alone (221). A phase II trial of monalizumab plus cetuximab in SCCHN patients showed a 31% ORR, with the most common AEs being fatigue (17%), pyrexia (13%), and headache (10%) (221). These results led to an ongoing phase III (INTERLINK-1) trial for RM HNSCC patients who underwent prior ICI- and platinum-based chemotherapy treatments. Although monalizumab has not yet gained FDA approval, it has shown benefit in enhancing the effects of other ICIs. However, future studies are needed to carefully define the suitable tumor types and to determine which type of combination therapy provides the optimal benefits.

In several preclinical cancer models, TIM-3 blockade displayed a slight advantage in terms of efficacy, especially when combined with PD-1 blockade (60, 224). Several-in-human Phase I/II trials have been initiated for numerous TIM-3 antibodies in patients with solid tumors or lymphoma (225). Most of them have been tested in conjunction with anti-PD-1/PD-L1 mAbs. TSR-022 (TIM-3 antibody, Tesaro) plus TSR-042 (anti-PD-1 antibody) demonstrated improved antitumor immunity in NSCLC patients who failed to respond to anti-PD-1 treatment alone previously (226). A SCLC patient who received LY3321367 (a novel TIM-3 mAb) 1200 mg Q2W as a single agent also achieved PR (227). All in all, these early data suggest that blocking TIM-3 alone or together with PD-1/PD-L1 is generally safe, well tolerated, and provides a slight advantage in efficacy compared to anti-PD-1/PD-L1 therapies alone.

### Combinatorial use of immune checkpoint blockade and other drug therapies

Despite the PD-1/PD-L1 pathway being recognized as a key checkpoint in the immune response of various cancer types, most patients do not benefit from PD-1/PD-L1 pathway blocking therapies due to primary or acquired resistance (228). The immune system can paradoxically suppress or promote cancer development and growth. Therefore, removing or blocking the factors that promote immune tolerance and boost anti-tumor immunity may facilitate better responses to anti-PD-1/PD-L1 therapy. Based on this conception, combinations of anti-PD-1/PD-L1 mAbs with chemotherapies, targeted therapies, or CTLA4 blockade have been tested clinically and some regimes become the standard of care for several cancer types including metastatic melanoma, kidney, lung, head and neck, triple negative breast (TNB), and liver cancers. Herein, we will briefly discuss the progress that has made with dual combination of ICIs and alternative therapeutic regimes (**Table 1**).

#### Combinatorial targeting of the CTLA4 and PD-1/PD-L1 pathways

Although CTLA4 and PD-1 are both immune checkpoints, they can suppress the activation of T-cells in non-redundant manners, and therefore dual blockade may work synergistically to enhance anti-tumor immune responses. Neoadjuvant ipilimumab plus nivolumab in patients with macroscopic stage III melanoma showed a 2-year estimated recurrence-free survival rate of 84% in all patients, a 97% estimated survival rate in patients who achieved a pathologic response, and a 36% estimated survival rate in non-responding patients (229). In addition to melanoma, many neoadjuvant ipilimumab plus nivolumab trials have been conducted in solid tumors, and have demonstrated enhanced responses in advanced RCC (230), microsatellite high/deficient mismatch repair colorectal cancer (mCRC MSI-H/dMMR) (231), HCC (232), NSCLC [at 12 months (233) and 24 months (234)], and malignant pleural mesothelioma (MPM) (235). Based on these clinical trials, the FDA has approved the combination of ipilimumab and nivolumab for the six cancer types mentioned above.

The combination of tremelimumab and durvalumab are being tested in several phase 3 trials (236–248). Although some results were unsatisfactory, this combination significantly improved OS in patients with advanced, unresectable HCC and NSCLC patients who had a high tumor mutation burden (TMB) (248, 249), showing the importance of selecting appropriate treatment regimens for specific patient subsets.

#### Combinatorial use of anti-PD-1/PD-L1 and other ICIs

Early in 2022, the FDA approved a fixed-dose combination of nivolumab and relatlimab for the treatment of unresectable or metastatic melanoma based on a phase II/III study (NCT03470922) (220). Data from the clinical trial showed a significantly superior PFS (median 10.2 months) in the combination group over nivolumab alone (4.6 months, p=0.0055). Combination therapy also reduced risk of death by 20% and improved OS rates versus nivolumab, although it did not reach statistical significance. The outcomes of patients with features often associated with poor prognosis such as high tumor burden, visceral metastases, increased serum LDH levels, or mucosal or acral melanoma, were improved in the dual-inhibition group compared with nivolumab alone (220). The safety profile of this combination was generally manageable and no novel or unexpected safety concerns were noted, although grade 3 or 4 TRAEs (such as fatigue, hepatitis, and adrenal insufficiency) were more frequently noted in patients receiving relatlimab-nivolumab than those receiving nivolumab (250). However, the efficacy of combined therapy in real-world patient needs to be further investigated since some populations of patients are often excluded from clinical trials, including patients with untreated or active brain metastases or with certain melanoma subtypes (e.g., uveal melanoma).

#### Combinatorial use of anti-PD-1/PD-L1 blockade and chemotherapy

Chemotherapeutic drugs slow tumor growth primarily by inhibiting DNA replication, interfering with cellular metabolism, inducing cell cycle arrest, or inhibiting microtubule assembly (251). In addition, some cytotoxic drugs such as oxaliplatin and anthracyclines can induce cell death of immunogenic cells, thereby stimulating anti-tumor immune responses (252, 253). Based on these effects, chemotherapeutic drugs may be suitable partners for combinatorial administration with anti-PD-1/PD-L1 to achieve rapid and long-term control of cancers. A large number of clinical trials have tested the efficacy and safety of chemotherapy plus anti-PD-1/PD-L1, with FDA approved combinations summarized in **Supplementary Table 1**. In general, pembrolizumab combined with chemotherapy has had great clinical benefit for a wide range of cancer indications, followed by atezolizumab combined with chemotherapy.

#### Combinatorial use of anti-PD-1/PD-L1 blockade and angiogenesis inhibitors

The hypoxic tumor microenvironment (TME) leads to the upregulation of vascular endothelial growth factor (VEGF) and angiopoietin 2 (ANGPT2), which are key mediators in angiogenesis (254). By inhibiting these pro-angiogenic pathways, angiogenesis inhibitors can promote vascular normalization, restore oxygenation within the typically hypoxic TME, improve perfusion and oxygenation in tumors, and enhance the delivery of antitumor drugs (255, 256). Several tyrosine kinase inhibitors that target the VEGF-signalling axis have been approved in the United States and European Union, including sorafenib, sunitinib, pazopanib, axitinib, lenvatinib, and cabozantinib (257).

The FDA recently approved the use of nivolumab plus cabozantinib, pembrolizumab/avelumab plus axitinib, and pembrolizumab plus lenvatinib in advanced RCC; pembrolizumab plus lenvatinib in advanced endometrial carcinoma; atezolizumab plus bevacizumab (anti-VEGF mAb) in unresectable or metastatic HCC; and atezolizumab plus bevacizumab and chemotherapy in non-squamous and metastatic NSCLC based on the results of these clinical trials (258–264).

#### Adverse effects of ICIs

With the use of ICIs for cancer treatment rising, several side effects associated with treatment have raised concerns. Compared to treatment with standard chemotherapy or other biologics, ICIs have a different toxicity profile and most of these AEs are caused by aberrant immune responses against normal self-tissues due to impaired self-tolerance from the loss of T-cell inhibition, a phenomenon known as immune-related adverse events (irAEs) (265). Three drugs (ipilimumab, nivolumab and pembrolizumab) were reported to account for nearly 60% of reported irAEs in patients receiving immune checkpoint blockade therapies (266).

IrAEs can affect any organ system, most commonly the skin, colon and endocrine systems (267). The onset time usually ranges from a few days after treatment initiation to ≥1 year after completion of treatment, with a median time of 2–16 weeks (268–270). Most irAEs occurring soon after administration (3 weeks after initiation of therapy) of CTLA4 and PD-1 inhibitors typically involves the skin (271). Serious and often life-threatening AEs include pneumonitis and colitis (272). In general, there are fewer AEs associated with anti-PD-1/PD-L1 drugs than CTLA4 inhibitors (273), potentially due to their different mechanisms of action. CTLA4 is expressed on T cells (including Tregs), and its activation primarily suppresses the immune response in the early stages of T cell activation in lymphoid tissues. As CTLA4 blockade of Tregs would also result in the loss of immune suppression, CTLA4 inhibition would result in widespread, nonspecific activation of immune response that might explain the broad spectrum of AEs (274, 275). In contrast, PD-1 inhibitors target T cells more specifically in the tumor microenvironment and tissues, resulting in a more restricted spectrum of AEs (274, 276).

Colitis and hypophysitis often occur in patients receiving CTLA4 inhibitors, while less common AEs (pneumonitis and thyroiditis) may occur in patients using drugs targeting PD-1/PD-L1 pathway (271, 277, 278). A meta-analysis of the irAEs of individual ICIs revealed that the most common side-effects associated with ipilimumab included dermatological, gastrointestinal, and renal toxicities; and side-effects associated with nivolumab often involved endocrine toxicities. Similarly, side-effects associated with pembrolizumab included arthralgia, pneumonitis, and hepatotoxicity; and irAEs associated with atezolizumab included hypothyroidism (279). Most AEs resolved without intervention or responded to appropriate treatment (280). Severe irAEs usually require the treatment of systemic glucocorticoids or other immunosuppressive medicines, such as anti-TNF blockers.

The combination therapy such as dual PD-1/PD-L1 and CTLA4 blockade can also lead to severe irAEs such as colitis, pneumonitis, hypophysitis, and thyroiditis (267). Therefore, in these combined regimens, the dose of ipilimumab is usually reduced, which may impair the efficacy of the combination therapy (281). In a preclinical study, prophylactic TNF blockade reduced the toxicity of dual PD-1/PD-L1 and CTLA4 blocking antibodies (282). When ICIs are combined with chemotherapies, the reported irAEs were consistent with those of each drug (283, 284). In order to minimize the potential toxicity of dual PD-1/PD-L1 and CTLA4 blockade, Dovedi et al. developed a bispecific anti–PD-1/CTLA4 antibody, MEDI5752 (285, 286). The antibody could inhibit the signal transduction through PD-1 axis and preferentially block CTLA4 on activated PD-1^+^ T cells over PD-1^-^ T cells, which means that CTLA4 is inhibited only in T cell populations that have already been exposed to the antigen. Therefore, the additive toxicity of this bispecific antibody is reduced compared with traditional dual blockers (285). In an ongoing in-human study in patients with advanced solid tumors, one patient with gastric cancer in whom five prior lines of chemotherapy had failed showed PR with 60% tumor reduction, and another patient with treatment-naïve renal clear cell carcinoma had PR with 68% tumor reduction. Both of the patients had manageable toxicity (285). However, more clinical studies are needed to determine the safety and efficacy of this bispecific agent or other combination therapies.

## Potential biomarkers of ICI response

We are now in an era of innovation in cancer immunotherapy that is transforming the field of clinical oncology. Sustained durable responses from ICIs provide new hope as a treatment option for patients previously diagnosed with terminal illnesses. However, only a small percentage of tumors are responsive to these therapies and the overall response rates are low. There might be several reasons for this (1). The corresponding TMB. High TMB accompanied by elevated neoantigen expression might induce cytotoxic responses against tumor cells (287, 288). Thus tumors with low TMB and poor immunogenicity such as prostate and pancreatic cancers would be more resistant to ICIs (287) (2). Intratumor heterogeneity may lead to the selection of subclones that lack neoantigen expression and confer resistance to immune therapies (289, 290) (3). Genetic instability such as alterations in DNA-mismatch repair genes can increase immunogenicity and enhance response to ICIs (291, 292). For example, melanoma patients responded better to anti-PD-1 therapy when their tumor cells were rich in mutations in BRCA2, a gene important for homologous recombination in DNA repair (293). Mutation of B2M was also reported to be associated with initial resistance to anti PD-1 therapy in melanoma patients (294, 295) (4). Potential biomarkers may predict the response to ICIs. For example, the greater the number of tumor-infiltrating lymphocytes (TIL) in the tumor microenvironment, the better the anti-tumor effect (296); and tumors manifesting PD-L1 overexpression usually portend clinical outcomes superior to those of ICI compared with those with lower levels of ligand (297) (5). Complex interactions of immune cells and cytokines in the TME. For example, Tregs promote self-tolerance by inhibiting the function of Teff through inhibitory cytokines and direct contacts (298), and in some cancers, the infiltration of Tregs suggest an immunosuppressive environment (299). Myeloid-derived suppressor cells (MDSCs) in the TME can promote tumor growth and immune evasion (300, 301) (6). Alterations to a patient’s gut microbiome have also been associated with response or resistance to immune-checkpoint blockade, although the exact mechanism remains unknown (302) (7). The presence of tertiary lymphoid structures (TLS) might indicate improved survival in some tumor types (303, 304). Higher densities of TLS were associated with increased tumor-infiltrating CD8+ T cells density and also with an activated and cytotoxic immune signature (304–306). The existence of mature TLS was associated with improved outcomes in cancer patients treated with ICIs, regardless of their PD-L1 status and tumor-infiltrating CD8+ T-cell level after adjustment (307).

There are also other factors that could influence the anti-tumor effect of ICIs, including T-cell exhaustion (308), chromatin remodeling (309), and upregulation of alternative negative immune-checkpoint molecules (310). Resolving these issues is posited to elevate the efficacy of ICIs to a higher level. For example, personalized vaccines composed of an individual patient’s tumor neoantigens might be taken up and presented by activated APCs, leading to the activation of specific T cells that target these neoantigens, and thereby enhancing the efficacy of PD-1/PD-L1 inhibitors (311). Targeting other immunomodulatory cells or cytokines such as MDSCs, anti-TGF-β, and PI3Kγ are currently in clinical trials (311, 312). Since some results are not promising, we still need to advance our overall understanding of the molecular biology of tumors.

In addition, it is necessary to explore how to better predict patient responses to improve clinical outcomes. Although the expression of PD-L1 on tumor cells, TIL status, and the assessment of mutational burden are presently used markers (290, 313–317), they often have poor specificity and sensitivity. Accurate prediction using biomarkers therefore remains a major clinical challenge and necessitates further investigation due to the complexity of antitumor immune responses and the heterogeneity among patients and tumors.

## Conclusions and perspective

The therapeutic landscape of oncology has been revolutionized with the advent of immunotherapies targeting immune checkpoints. A growing number of ICIs have been approved to treat different cancers. They can be used alone or in combination, or combined with other chemotherapies, vaccines and tumor immunotherapies, etc. Although these ICIs can bring survival benefits, their response rates and PFS are not high, and they also have a variety of adverse effects. A better understanding of the regulatory pathways of immune checkpoints will improve the success and efficacy of ICIs.

With in-depth investigations into immunotherapy, more novel immune checkpoints, regulators, and receptor-ligand pathways between tumor and host immune cells will continue to be discovered and further advance our understanding of the mechanisms leading to tumor immune evasion or resistance. Individualized therapeutic strategies based on a patient’s genetic background may suppress overactive T-cell responses more robustly, and thereby assist in patient selection. Further research is urgently needed to elucidate the mechanisms underlying tumor biology and immunotherapy, how one ICI affects another, and the development of novel sequential treatment options, so as to facilitate the successful use of ICIs in treating cancer patients.

## Author contributions

YW and QZ (4^th^ author) conceived and designed the research. LY and MS prepared the draft. QZ (3^rd^ author) prepared the tables. QZ (3^rd^ author) prepared the figure. YW and QZ (4^th^ author) reviewed and edited the manuscript. All authors read and approved the final manuscript.

## Funding

This study was sponsored by an grant from the National Science Foundation of China (81802504, 81872207), a grant from the Sichuan Medical Association (Q19037), grants of Sichuan Science and Technology Bureau (2019YFS0439, 2020JDJQ0067, 2021YJ0564, 2022YFH0005), and a grant from the Chengdu Science and Technology Bureau (2021-YF05-00225-SN).

## Conflict of interest

The authors declare that the research was conducted in the absence of any commercial or financial relationships that could be construed as a potential conflict of interest.

## Publisher’s note

All claims expressed in this article are solely those of the authors and do not necessarily represent those of their affiliated organizations, or those of the publisher, the editors and the reviewers. Any product that may be evaluated in this article, or claim that may be made by its manufacturer, is not guaranteed or endorsed by the publisher.

## References

[1] Dunn GP Bruce AT Ikeda H Old LJ Schreiber RD. Cancer immunoediting: from immunosurveillance to tumor escape. Nat Immunol (2002) 3(11):991–8. doi: 10.1038/ni1102-991 12407406

[2] Burnet FM. The concept of immunological surveillance. Prog Exp Tumor Res (1970) 13:1–27. doi: 10.1159/000386035 4921480

[3] Burnet M. Cancer: a biological approach. III. viruses associated with neoplastic conditions. IV. practical applications. Br Med J (1957) 1(5023):841–7. doi: 10.1136/bmj.1.5023.841 PMC1973618 13413231

[4] Zhang Y Zhang X Li W Du Y Hu W Zhao J. Biomarkers and risk factors for the early prediction of immune-related adverse events: a review. Hum Vaccin Immunother (2022) 18(1):2018894. doi: 10.1080/21645515.2021.2018894 35108160 PMC8986173

[5] Haddad AF Young JS Gill S Aghi MK. Resistance to immune checkpoint blockade: Mechanisms, counter-acting approaches, and future directions. Semin Cancer Biol (2022), S1044–579X(22)00046-3. doi: 10.1016/j.semcancer.2022.02.019 PMC9458771 35276342

[6] Zappasodi R Merghoub T Wolchok JD. Emerging concepts for immune checkpoint blockade-based combination therapies. Cancer Cell (2018) 33(4):581–98. doi: 10.1016/j.ccell.2018.03.005 PMC5896787 29634946

[7] Tu L Guan R Yang H Zhou Y Hong W Ma L. Assessment of the expression of the immune checkpoint molecules PD-1, CTLA4, TIM-3 and LAG-3 across different cancers in relation to treatment response, tumor-infiltrating immune cells and survival. Int J Cancer (2020) 147(2):423–39. doi: 10.1002/ijc.32785 31721169

[8] Rowshanravan B Halliday N Sansom DM. CTLA-4: a moving target in immunotherapy. Blood (2018) 131(1):58–67. doi: 10.1182/blood-2017-06-741033 29118008 PMC6317697

[9] McCoy KD Le Gros G. The role of CTLA-4 in the regulation of T cell immune responses. Immunol Cell Biol (1999) 77(1):1–10. doi: 10.1046/j.1440-1711.1999.00795.x 10101680

[10] Alegre ML Noel PJ Eisfelder BJ Chuang E Clark MR Reiner SL. Regulation of surface and intracellular expression of CTLA4 on mouse T cells. J Immunol (1996) 157(11):4762–70. 8943377

[11] Chikuma S. CTLA-4, an essential immune-checkpoint for T-cell activation. Curr Top Microbiol Immunol (2017) 410:99–126. doi: 10.1007/82_2017_61 28900679

[12] Tai X Van Laethem F Pobezinsky L Guinter T Sharrow SO Adams A. Basis of CTLA-4 function in regulatory and conventional CD4(+) T cells. Blood (2012) 119(22):5155–63. doi: 10.1182/blood-2011-11-388918 PMC3369608 22403258

[13] Qureshi OS Zheng Y Nakamura K Attridge K Manzotti C Schmidt EM. Trans-endocytosis of CD80 and CD86: a molecular basis for the cell-extrinsic function of CTLA-4. Science (2011) 332(6029):600–3. doi: 10.1126/science.1202947 PMC3198051 21474713

[14] Walunas TL Lenschow DJ Bakker CY Linsley PS Freeman GJ Green JM. CTLA-4 can function as a negative regulator of T cell activation. Immunity (1994) 1(5):405–13. doi: 10.1016/1074-7613(94)90071-x 7882171

[15] Schneider H Downey J Smith A Zinselmeyer BH Rush C Brewer JM. Reversal of the TCR stop signal by CTLA-4. Science (2006) 313(5795):1972–5. doi: 10.1126/science.1131078 16931720

[16] Corse E Allison JP. Cutting edge: CTLA-4 on effector T cells inhibits in trans. J Immunol (2012) 189(3):1123–7. doi: 10.4049/jimmunol.1200695 22753941

[17] Naimi A Mohammed RN Raji A Chupradit S Yumashev AV Suksatan W. Tumor immunotherapies by immune checkpoint inhibitors (ICIs); the pros and cons. Cell Commun Signal (2022) 20(1):44. doi: 10.1186/s12964-022-00854-y 35392976 PMC8991803

[18] Tivol EA Borriello F Schweitzer AN Lynch WP Bluestone JA Sharpe AH. Loss of CTLA-4 leads to massive lymphoproliferation and fatal multiorgan tissue destruction, revealing a critical negative regulatory role of CTLA-4. Immunity (1995) 3(5):541–7. doi: 10.1016/1074-7613(95)90125-6 7584144

[19] Valk E Rudd CE Schneider H. CTLA-4 trafficking and surface expression. Trends Immunol (2008) 29(6):272–9. doi: 10.1016/j.it.2008.02.011 PMC4186961 18468488

[20] Shiratori T Miyatake S Ohno H Nakaseko C Isono K Bonifacino JS. Tyrosine phosphorylation controls internalization of CTLA-4 by regulating its interaction with clathrin-associated adaptor complex AP-2. Immunity (1997) 6(5):583–9. doi: 10.1016/s1074-7613(00)80346-5 9175836

[21] Zhang Y Allison JP. Interaction of CTLA-4 with AP50, a clathrin-coated pit adaptor protein. Proc Natl Acad Sci U.S.A. (1997) 94(17):9273–8. doi: 10.1073/pnas.94.17.9273 PMC23153 9256472

[22] Greenwald RJ Boussiotis VA Lorsbach RB Abbas AK Sharpe AH. CTLA-4 regulates induction of anergy *in vivo* . Immunity (2001) 14(2):145–55. doi: 10.1016/s1074-7613(01)00097-8 11239447

[23] Sojka DK Hughson A Fowell DJ. CTLA-4 is required by CD4+CD25+ treg to control CD4+ T-cell lymphopenia-induced proliferation. Eur J Immunol (2009) 39(6):1544–51. doi: 10.1002/eji.200838603 PMC2835300 19462377

[24] Wing K Onishi Y Prieto-Martin P Yamaguchi T Miyara M Fehervari Z. CTLA-4 control over Foxp3+ regulatory T cell function. Science (2008) 322(5899):271–5. doi: 10.1126/science.1160062 18845758

[25] Tang Q Boden EK Henriksen KJ Bour-Jordan H Bi M Bluestone JA. Distinct roles of CTLA-4 and TGF-beta in CD4+CD25+ regulatory T cell function. Eur J Immunol (2004) 34(11):2996–3005. doi: 10.1002/eji.200425143 15468055

[26] Bengsch F Knoblock DM Liu A McAllister F Beatty GL. CTLA-4/CD80 pathway regulates T cell infiltration into pancreatic cancer. Cancer Immunol Immunother (2017) 66(12):1609–17. doi: 10.1007/s00262-017-2053-4 PMC5677559 28856392

[27] Contardi E Palmisano GL Tazzari PL Martelli AM Fala F Fabbi M. CTLA-4 is constitutively expressed on tumor cells and can trigger apoptosis upon ligand interaction. Int J Cancer (2005) 117(4):538–50. doi: 10.1002/ijc.21155 15912538

[28] Liu Y Zheng P. How does an anti-CTLA-4 antibody promote cancer immunity? Trends Immunol (2018) 39(12):953–6. doi: 10.1016/j.it.2018.10.009 PMC6589807 30497614

[29] Ha D Tanaka A Kibayashi T Tanemura A Sugiyama D Wing JB. Differential control of human treg and effector T cells in tumor immunity by fc-engineered anti-CTLA-4 antibody. Proc Natl Acad Sci U.S.A. (2019) 116(2):609–18. doi: 10.1073/pnas.1812186116 PMC6329979 30587582

[30] Sharma A Subudhi SK Blando J Scutti J Vence L Wargo J. Anti-CTLA-4 immunotherapy does not deplete FOXP3(+) regulatory T cells (Tregs) in human cancers. Clin Cancer Res (2019) 25(4):1233–8. doi: 10.1158/1078-0432.CCR-18-0762 PMC6348141 30054281

[31] Ishida Y Agata Y Shibahara K Honjo T. Induced expression of PD-1, a novel member of the immunoglobulin gene superfamily, upon programmed cell death. EMBO J (1992) 11(11):3887–95. 10.1002/j.1460-2075.1992.tb05481.x PMC556898 1396582

[32] De Silva P Aiello M Gu-Trantien C Migliori E Willard-Gallo K Solinas C. Targeting CTLA-4 in cancer: Is it the ideal companion for PD-1 blockade immunotherapy combinations? Int J Cancer (2021) 149(1):31–41. doi: 10.1002/ijc.33415 33252786

[33] Mizuno R Sugiura D Shimizu K Maruhashi T Watada M Okazaki IM. PD-1 primarily targets TCR signal in the inhibition of functional T cell activation. Front Immunol (2019) 10:630. doi: 10.3389/fimmu.2019.00630 31001256 PMC6455061

[34] Honda T Egen JG Lammermann T Kastenmuller W Torabi-Parizi P Germain RN. Tuning of antigen sensitivity by T cell receptor-dependent negative feedback controls T cell effector function in inflamed tissues. Immunity (2014) 40(2):235–47. doi: 10.1016/j.immuni.2013.11.017 PMC4792276 24440150

[35] Andrews LP Yano H Vignali DAA. Inhibitory receptors and ligands beyond PD-1, PD-L1 and CTLA-4: breakthroughs or backups. Nat Immunol (2019) 20(11):1425–34. doi: 10.1038/s41590-019-0512-0 31611702

[36] Esch KJ Juelsgaard R Martinez PA Jones DE Petersen CA. Programmed death 1-mediated T cell exhaustion during visceral leishmaniasis impairs phagocyte function. J Immunol (2013) 191(11):5542–50. doi: 10.4049/jimmunol.1301810 PMC3896087 24154626

[37] Lo B Abdel-Motal UM. Lessons from CTLA-4 deficiency and checkpoint inhibition. Curr Opin Immunol (2017) 49:14–9. doi: 10.1016/j.coi.2017.07.014 28806575

[38] Nishimura H Nose M Hiai H Minato N Honjo T. Development of lupus-like autoimmune diseases by disruption of the PD-1 gene encoding an ITIM motif-carrying immunoreceptor. Immunity (1999) 11(2):141–51. doi: 10.1016/s1074-7613(00)80089-8 10485649

[39] Nishimura H Honjo T Minato N. Facilitation of beta selection and modification of positive selection in the thymus of PD-1-deficient mice. J Exp Med (2000) 191(5):891–8. doi: 10.1084/jem.191.5.891 PMC2195853 10704469

[40] Buchbinder EI Desai A. CTLA-4 and PD-1 pathways: Similarities, differences, and implications of their inhibition. Am J Clin Oncol (2016) 39(1):98–106. doi: 10.1097/COC.0000000000000239 26558876 PMC4892769

[41] Kahler KC Hauschild A. Treatment and side effect management of CTLA-4 antibody therapy in metastatic melanoma. J Dtsch Dermatol Ges (2011) 9(4):277–86. doi: 10.1111/j.1610-0387.2010.07568.x 21083648

[42] Aksoylar HI Boussiotis VA. PD-1(+) treg cells: a foe in cancer immunotherapy? Nat Immunol (2020) 21(11):1311–2. doi: 10.1038/s41590-020-0801-7 PMC10754338 32973361

[43] Francisco LM Salinas VH Brown KE Vanguri VK Freeman GJ Kuchroo VK. PD-L1 regulates the development, maintenance, and function of induced regulatory T cells. J Exp Med (2009) 206(13):3015–29. doi: 10.1084/jem.20090847 PMC2806460 20008522

[44] Pan T Liu Z Yin J Zhou T Liu J Qu H. Notch signaling pathway was involved in regulating programmed cell death 1 expression during sepsis-induced immunosuppression. Mediators Inflammation (2015) 2015:539841. doi: 10.1155/2015/539841 PMC4430661 26063974

[45] Amarnath S Mangus CW Wang JC Wei F He A Kapoor V. The PDL1-PD1 axis converts human TH1 cells into regulatory T cells. Sci Transl Med (2011) 3(111):111ra120. doi: 10.1126/scitranslmed.3003130 PMC3235958 22133721

[46] Kamada T Togashi Y Tay C Ha D Sasaki A Nakamura Y. PD-1(+) regulatory T cells amplified by PD-1 blockade promote hyperprogression of cancer. Proc Natl Acad Sci U.S.A. (2019) 116(20):9999–10008. doi: 10.1073/pnas.1822001116 31028147 PMC6525547

[47] Zhang Y Liu Z Tian M Hu X Wang L Ji J. The altered PD-1/PD-L1 pathway delivers the 'one-two punch' effects to promote the Treg/Th17 imbalance in pre-eclampsia. Cell Mol Immunol (2018) 15(7):710–23. doi: 10.1038/cmi.2017.70 PMC6123412 28890543

[48] Kumagai S Togashi Y Kamada T Sugiyama E Nishinakamura H Takeuchi Y. The PD-1 expression balance between effector and regulatory T cells predicts the clinical efficacy of PD-1 blockade therapies. Nat Immunol (2020) 21(11):1346–58. doi: 10.1038/s41590-020-0769-3 32868929

[49] Das M Zhu C Kuchroo VK. Tim-3 and its role in regulating anti-tumor immunity. Immunol Rev (2017) 276(1):97–111. doi: 10.1111/imr.12520 28258697 PMC5512889

[50] Huang YH Zhu C Kondo Y Anderson AC Gandhi A Russell A. CEACAM1 regulates TIM-3-mediated tolerance and exhaustion. Nature (2015) 517(7534):386–90. doi: 10.1038/nature13848 PMC4297519 25363763

[51] Sanchez-Fueyo A Tian J Picarella D Domenig C Zheng XX Sabatos CA. Tim-3 inhibits T helper type 1-mediated auto- and alloimmune responses and promotes immunological tolerance. Nat Immunol (2003) 4(11):1093–101. doi: 10.1038/ni987 14556005

[52] Zhu C Anderson AC Schubart A Xiong H Imitola J Khoury SJ. The Tim-3 ligand galectin-9 negatively regulates T helper type 1 immunity. Nat Immunol (2005) 6(12):1245–52. doi: 10.1038/ni1271 16286920

[53] Sabatos CA Chakravarti S Cha E Schubart A Sanchez-Fueyo A Zheng XX. Interaction of Tim-3 and Tim-3 ligand regulates T helper type 1 responses and induction of peripheral tolerance. Nat Immunol (2003) 4(11):1102–10. doi: 10.1038/ni988 14556006

[54] Dardalhon V Anderson AC Karman J Apetoh L Chandwaskar R Lee DH. Tim-3/galectin-9 pathway: regulation of Th1 immunity through promotion of CD11b+Ly-6G+ myeloid cells. J Immunol (2010) 185(3):1383–92. doi: 10.4049/jimmunol.0903275 PMC2925247 20574007

[55] Li H Wu K Tao K Chen L Zheng Q Lu X. Tim-3/galectin-9 signaling pathway mediates T-cell dysfunction and predicts poor prognosis in patients with hepatitis b virus-associated hepatocellular carcinoma. Hepatology (2012) 56(4):1342–51. doi: 10.1002/hep.25777 22505239

[56] Monney L Sabatos CA Gaglia JL Ryu A Waldner H Chernova T. Th1-specific cell surface protein Tim-3 regulates macrophage activation and severity of an autoimmune disease. Nature (2002) 415(6871):536–41. doi: 10.1038/415536a 11823861

[57] Liu Z McMichael EL Shayan G Li J Chen K Srivastava R. Novel effector phenotype of Tim-3(+) regulatory T cells leads to enhanced suppressive function in head and neck cancer patients. Clin Cancer Res (2018) 24(18):4529–38. doi: 10.1158/1078-0432.CCR-17-1350 PMC6139056 29712685

[58] Gautron AS Dominguez-Villar M de Marcken M Hafler DA. Enhanced suppressor function of TIM-3+ FoxP3+ regulatory T cells. Eur J Immunol (2014) 44(9):2703–11. doi: 10.1002/eji.201344392 PMC4165702 24838857

[59] Liu JF Wu L Yang LL Deng WW Mao L Wu H. Blockade of TIM3 relieves immunosuppression through reducing regulatory T cells in head and neck cancer. J Exp Clin Cancer Res (2018) 37(1):44. doi: 10.1186/s13046-018-0713-7 29506555 PMC5838931

[60] Sakuishi K Apetoh L Sullivan JM Blazar BR Kuchroo VK Anderson AC. Targeting Tim-3 and PD-1 pathways to reverse T cell exhaustion and restore anti-tumor immunity. J Exp Med (2010) 207(10):2187–94. doi: 10.1084/jem.20100643 PMC2947065 20819927

[61] Liu J Zhang S Hu Y Yang Z Li J Liu X. Targeting PD-1 and Tim-3 pathways to reverse CD8 T-cell exhaustion and enhance ex vivo T-cell responses to autologous Dendritic/Tumor vaccines. J Immunother (2016) 39(4):171–80. doi: 10.1097/CJI.0000000000000122 27070448

[62] Fourcade J Sun Z Benallaoua M Guillaume P Luescher IF Sander C. Upregulation of Tim-3 and PD-1 expression is associated with tumor antigen-specific CD8+ T cell dysfunction in melanoma patients. J Exp Med (2010) 207(10):2175–86. doi: 10.1084/jem.20100637 PMC2947081 20819923

[63] Shi X Zhang X Li J Mo L Zhao H Zhu Y. PD-1 blockade enhances the antitumor efficacy of GM-CSF surface-modified bladder cancer stem cells vaccine. Int J Cancer (2018) 142(10):2106–17. doi: 10.1002/ijc.31219 29243219

[64] Zhang X Liu G Shi X Shi X Li J Mo L. Sequential administration of anti-PD-1 and anti-Tim-3 combined with an SA-GM-CSF-anchored vaccine overcomes adaptive immune resistance to reject established bladder cancer. J Cancer (2021) 12(7):2000–9. doi: 10.7150/jca.44769 PMC7974521 33753998

[65] Zhang X Chen H Li G Zhou X Shi Y Zou F. Increased Tim-3 expression on TILs during treatment with the anchored GM-CSF vaccine and anti-PD-1 antibodies is inversely correlated with response in prostate cancer. J Cancer (2020) 11(3):648–56. doi: 10.7150/jca.29705 PMC6959042 31942188

[66] Mimura K Kua LF Xiao JF Asuncion BR Nakayama Y Syn N. Combined inhibition of PD-1/PD-L1, lag-3, and Tim-3 axes augments antitumor immunity in gastric cancer-T cell coculture models. Gastric Cancer (2021) 24(3):611–23. doi: 10.1007/s10120-020-01151-8 PMC8065004 33611641

[67] Shi X Li CW Tan LC Wen SS Liao T Zhang Y. Immune Co-inhibitory receptors PD-1, CTLA-4, TIM-3, LAG-3, and TIGIT in medullary thyroid cancers: A Large cohort study. J Clin Endocrinol Metab (2021) 106(1):120–32. doi: 10.1210/clinem/dgaa701 33000173

[68] Jie HB Srivastava RM Argiris A Bauman JE Kane LP Ferris RL. Increased PD-1(+) and TIM-3(+) TILs during cetuximab therapy inversely correlate with response in head and neck cancer patients. Cancer Immunol Res (2017) 5(5):408–16. doi: 10.1158/2326-6066.CIR-16-0333 PMC5497750 28408386

[69] Triebel F Jitsukawa S Baixeras E Roman-Roman S Genevee C Viegas-Pequignot E. LAG-3, a novel lymphocyte activation gene closely related to CD4. J Exp Med (1990) 171(5):1393–405. doi: 10.1084/jem.171.5.1393 PMC2187904 1692078

[70] Huard B Gaulard P Faure F Hercend T Triebel F. Cellular expression and tissue distribution of the human LAG-3-encoded protein, an MHC class II ligand. Immunogenetics (1994) 39(3):213–7. doi: 10.1007/BF00241263 7506235

[71] Kisielow M Kisielow J Capoferri-Sollami G Karjalainen K. Expression of lymphocyte activation gene 3 (LAG-3) on b cells is induced by T cells. Eur J Immunol (2005) 35(7):2081–8. doi: 10.1002/eji.200526090 15971272

[72] Solinas C Migliori E De Silva P Willard-Gallo K. LAG3: The biological processes that motivate targeting this immune checkpoint molecule in human cancer. Cancers (Basel) (2019) 11(8):1213. doi: 10.3390/cancers11081213 PMC6721578 31434339

[73] Huard B Prigent P Tournier M Bruniquel D Triebel F. CD4/major histocompatibility complex class II interaction analyzed with CD4- and lymphocyte activation gene-3 (LAG-3)-Ig fusion proteins. Eur J Immunol (1995) 25(9):2718–21. doi: 10.1002/eji.1830250949 7589152

[74] Workman CJ Vignali DA. Negative regulation of T cell homeostasis by lymphocyte activation gene-3 (CD223). J Immunol (2005) 174(2):688–95. doi: 10.4049/jimmunol.174.2.688 15634887

[75] Grosso JF Kelleher CC Harris TJ Maris CH Hipkiss EL De Marzo A. LAG-3 regulates CD8+ T cell accumulation and effector function in murine self- and tumor-tolerance systems. J Clin Invest (2007) 117(11):3383–92. doi: 10.1172/JCI31184 PMC2000807 17932562

[76] Workman CJ Vignali DA. The CD4-related molecule, LAG-3 (CD223), regulates the expansion of activated T cells. Eur J Immunol (2003) 33(4):970–9. doi: 10.1002/eji.200323382 12672063

[77] Huang CT Workman CJ Flies D Pan X Marson AL Zhou G. Role of LAG-3 in regulatory T cells. Immunity (2004) 21(4):503–13. doi: 10.1016/j.immuni.2004.08.010 15485628

[78] Camisaschi C Casati C Rini F Perego M De Filippo A Triebel F. LAG-3 expression defines a subset of CD4(+)CD25(high)Foxp3(+) regulatory T cells that are expanded at tumor sites. J Immunol (2010) 184(11):6545–51. doi: 10.4049/jimmunol.0903879 20421648

[79] Gagliani N Magnani CF Huber S Gianolini ME Pala M Licona-Limon P. Coexpression of CD49b and LAG-3 identifies human and mouse T regulatory type 1 cells. Nat Med (2013) 19(6):739–46. doi: 10.1038/nm.3179 23624599

[80] Workman CJ Wang Y El Kasmi KC Pardoll DM Murray PJ Drake CG. LAG-3 regulates plasmacytoid dendritic cell homeostasis. J Immunol (2009) 182(4):1885–91. doi: 10.4049/jimmunol.0800185 PMC2675170 19201841

[81] Andreae S Piras F Burdin N Triebel F. Maturation and activation of dendritic cells induced by lymphocyte activation gene-3 (CD223). J Immunol (2002) 168(8):3874–80. doi: 10.4049/jimmunol.168.8.3874 11937541

[82] Hu S Liu X Li T Li Z Hu F. LAG3 (CD223) and autoimmunity: Emerging evidence. J Autoimmun (2020) 112:102504. doi: 10.1016/j.jaut.2020.102504 32576412

[83] Long L Zhang X Chen F Pan Q Phiphatwatchara P Zeng Y. The promising immune checkpoint LAG-3: from tumor microenvironment to cancer immunotherapy. Genes Cancer (2018) 9(5-6):176–89. doi: 10.18632/genesandcancer.180 PMC6305110 30603054

[84] Anderson AC Joller N Kuchroo VK. Lag-3, Tim-3, and TIGIT: Co-inhibitory receptors with specialized functions in immune regulation. Immunity (2016) 44(5):989–1004. doi: 10.1016/j.immuni.2016.05.001 27192565 PMC4942846

[85] Burugu S Gao D Leung S Chia SK Nielsen TO. LAG-3+ tumor infiltrating lymphocytes in breast cancer: clinical correlates and association with PD-1/PD-L1+ tumors. Ann Oncol (2017) 28(12):2977–84. doi: 10.1093/annonc/mdx557 29045526

[86] Matsuzaki J Gnjatic S Mhawech-Fauceglia P Beck A Miller A Tsuji T. Tumor-infiltrating NY-ESO-1-specific CD8+ T cells are negatively regulated by LAG-3 and PD-1 in human ovarian cancer. Proc Natl Acad Sci U.S.A. (2010) 107(17):7875–80. doi: 10.1073/pnas.1003345107 PMC2867907 20385810

[87] He Y Yu H Rozeboom L Rivard CJ Ellison K Dziadziuszko R. LAG-3 protein expression in non-small cell lung cancer and its relationship with PD-1/PD-L1 and tumor-infiltrating lymphocytes. J Thorac Oncol (2017) 12(5):814–23. doi: 10.1016/j.jtho.2017.01.019 28132868

[88] Huang RY Eppolito C Lele S Shrikant P Matsuzaki J Odunsi K. LAG3 and PD1 co-inhibitory molecules collaborate to limit CD8+ T cell signaling and dampen antitumor immunity in a murine ovarian cancer model. Oncotarget (2015) 6(29):27359–77. doi: 10.18632/oncotarget.4751 PMC4694995 26318293

[89] Woo SR Turnis ME Goldberg MV Bankoti J Selby M Nirschl CJ. Immune inhibitory molecules LAG-3 and PD-1 synergistically regulate T-cell function to promote tumoral immune escape. Cancer Res (2012) 72(4):917–27. doi: 10.1158/0008-5472.CAN-11-1620 PMC3288154 22186141

[90] Zelba H Bedke J Hennenlotter J Mostbock S Zettl M Zichner T. PD-1 and LAG-3 dominate checkpoint receptor-mediated T-cell inhibition in renal cell carcinoma. Cancer Immunol Res (2019) 7(11):1891–9. doi: 10.1158/2326-6066.CIR-19-0146 31484656

[91] Hafler JP Maier LM Cooper JD Plagnol V Hinks A Simmonds MJ. CD226 Gly307Ser association with multiple autoimmune diseases. Genes Immun (2009) 10(1):5–10. doi: 10.1038/gene.2008.82 18971939 PMC2635550

[92] Yu X Harden K Gonzalez LC Francesco M Chiang E Irving B. The surface protein TIGIT suppresses T cell activation by promoting the generation of mature immunoregulatory dendritic cells. Nat Immunol (2009) 10(1):48–57. doi: 10.1038/ni.1674 19011627

[93] Liu XG Hou M Liu Y. TIGIT, a novel therapeutic target for tumor immunotherapy. Immunol Invest (2017) 46(2):172–82. doi: 10.1080/08820139.2016.1237524 27819527

[94] Lozano E Dominguez-Villar M Kuchroo V Hafler DA. The TIGIT/CD226 axis regulates human T cell function. J Immunol (2012) 188(8):3869–75. doi: 10.4049/jimmunol.1103627 PMC3324669 22427644

[95] Johnston RJ Comps-Agrar L Hackney J Yu X Huseni M Yang Y. The immunoreceptor TIGIT regulates antitumor and antiviral CD8(+) T cell effector function. Cancer Cell (2014) 26(6):923–37. doi: 10.1016/j.ccell.2014.10.018 25465800

[96] Kurtulus S Sakuishi K Ngiow SF Joller N Tan DJ Teng MW. TIGIT predominantly regulates the immune response *via* regulatory T cells. J Clin Invest (2015) 125(11):4053–62. doi: 10.1172/JCI81187 PMC4639980 26413872

[97] Joller N Lozano E Burkett PR Patel B Xiao S Zhu C. Treg cells expressing the coinhibitory molecule TIGIT selectively inhibit proinflammatory Th1 and Th17 cell responses. Immunity (2014) 40(4):569–81. doi: 10.1016/j.immuni.2014.02.012 PMC4070748 24745333

[98] Lucca LE Axisa PP Singer ER Nolan NM Dominguez-Villar M Hafler DA. TIGIT signaling restores suppressor function of Th1 tregs. JCI Insight (2019) 4(3):e124427. doi: 10.1172/jci.insight.124427 PMC6413794 30728325

[99] Stanietsky N Rovis TL Glasner A Seidel E Tsukerman P Yamin R. Mouse TIGIT inhibits NK-cell cytotoxicity upon interaction with PVR. Eur J Immunol (2013) 43(8):2138–50. doi: 10.1002/eji.201243072 PMC3863769 23677581

[100] Stanietsky N Simic H Arapovic J Toporik A Levy O Novik A. The interaction of TIGIT with PVR and PVRL2 inhibits human NK cell cytotoxicity. Proc Natl Acad Sci U.S.A. (2009) 106(42):17858–63. doi: 10.1073/pnas.0903474106 PMC2764881 19815499

[101] Zhang Q Bi J Zheng X Chen Y Wang H Wu W. Blockade of the checkpoint receptor TIGIT prevents NK cell exhaustion and elicits potent anti-tumor immunity. Nat Immunol (2018) 19(7):723–32. doi: 10.1038/s41590-018-0132-0 29915296

[102] Chauvin JM Pagliano O Fourcade J Sun Z Wang H Sander C. TIGIT and PD-1 impair tumor antigen-specific CD8(+) T cells in melanoma patients. J Clin Invest (2015) 125(5):2046–58. doi: 10.1172/JCI80445 PMC4463210 25866972

[103] Hung AL Maxwell R Theodros D Belcaid Z Mathios D Luksik AS. TIGIT and PD-1 dual checkpoint blockade enhances antitumor immunity and survival in GBM. Oncoimmunology (2018) 7(8):e1466769. doi: 10.1080/2162402X.2018.1466769 30221069 PMC6136875

[104] Josefsson SE Beiske K Blaker YN Forsund MS Holte H Ostenstad B. TIGIT and PD-1 mark intratumoral T cells with reduced effector function in b-cell non-Hodgkin lymphoma. Cancer Immunol Res (2019) 7(3):355–62. doi: 10.1158/2326-6066.CIR-18-0351 PMC6636339 30659053

[105] Xu D Zhao E Zhu C Zhao W Wang C Zhang Z. TIGIT and PD-1 may serve as potential prognostic biomarkers for gastric cancer. Immunobiology (2020) 225(3):151915. doi: 10.1016/j.imbio.2020.151915 32122675

[106] Yang ZZ Kim HJ Wu H Jalali S Tang X Krull JE. TIGIT expression is associated with T-cell suppression and exhaustion and predicts clinical outcome and anti-PD-1 response in follicular lymphoma. Clin Cancer Res (2020) 26(19):5217–31. doi: 10.1158/1078-0432.CCR-20-0558 32631956

[107] Wang XF Chen YJ Wang Q Ge Y Dai Q Yang KF. Distinct expression and inhibitory function of b and T lymphocyte attenuator on human T cells. Tissue Antigens (2007) 69(2):145–53. doi: 10.1111/j.1399-0039.2006.00710.x 17257317

[108] Ning Z Liu K Xiong H. Roles of BTLA in immunity and immune disorders. Front Immunol (2021) 12:654960. doi: 10.3389/fimmu.2021.654960 33859648 PMC8043046

[109] Xu X Hou B Fulzele A Masubuchi T Zhao Y Wu Z. PD-1 and BTLA regulate T cell signaling differentially and only partially through SHP1 and SHP2. J Cell Biol (2020) 219(6):e201905085. doi: 10.1083/jcb.201905085 32437509 PMC7265324

[110] Vendel AC Calemine-Fenaux J Izrael-Tomasevic A Chauhan V Arnott D Eaton DL. B and T lymphocyte attenuator regulates b cell receptor signaling by targeting syk and BLNK. J Immunol (2009) 182(3):1509–17. doi: 10.4049/jimmunol.182.3.1509 19155498

[111] Simon T Bromberg JS. BTLA(+) dendritic cells: The regulatory T cell force awakens. Immunity (2016) 45(5):956–8. doi: 10.1016/j.immuni.2016.10.030 27851922

[112] Yang C Chen Y Guo G Li H Cao D Xu H. Expression of b and T lymphocyte attenuator (BTLA) in macrophages contributes to the fulminant hepatitis caused by murine hepatitis virus strain-3. Gut (2013) 62(8):1204–13. doi: 10.1136/gutjnl-2012-302239 22637698

[113] Li S Zhang M Xiang F Zhao J Jiang C Zhu J. Dendritic cells expressing BTLA induces CD8+ T cell tolerance and attenuates the severity of diabetes. Vaccine (2011) 29(44):7747–51. doi: 10.1016/j.vaccine.2011.07.125 21827810

[114] Bourque J Hawiger D. The BTLA-HVEM-CD5 immunoregulatory axis-an instructive mechanism governing pTreg cell differentiation. Front Immunol (2019) 10:1163. doi: 10.3389/fimmu.2019.01163 31191536 PMC6541033

[115] Huarte E Jun S Rynda-Apple A Golden S Jackiw L Hoffman C. Regulatory T cell dysfunction acquiesces to BTLA+ regulatory b cells subsequent to oral intervention in experimental autoimmune encephalomyelitis. J Immunol (2016) 196(12):5036–46. doi: 10.4049/jimmunol.1501973 PMC4893905 27194787

[116] Song J Wu L. Friend or foe: Prognostic and immunotherapy roles of BTLA in colorectal cancer. Front Mol Biosci (2020) 7:148. doi: 10.3389/fmolb.2020.00148 32793631 PMC7385242

[117] Chen YL Lin HW Chien CL Lai YL Sun WZ Chen CA. BTLA blockade enhances cancer therapy by inhibiting IL-6/IL-10-induced CD19(high) b lymphocytes. J Immunother Cancer (2019) 7(1):313. doi: 10.1186/s40425-019-0744-4 31753019 PMC6868712

[118] Lan X Li S Gao H Nanding A Quan L Yang C. Increased BTLA and HVEM in gastric cancer are associated with progression and poor prognosis. Onco Targets Ther (2017) 10:919–26. doi: 10.2147/OTT.S128825 PMC5317317 28243127

[119] Li X Xu Z Cui G Yu L Zhang X. BTLA expression in stage I-III non-Small-Cell lung cancer and its correlation with PD-1/PD-L1 and clinical outcomes. Onco Targets Ther (2020) 13:215–24. doi: 10.2147/OTT.S232234 PMC6957103 32021268

[120] Fourcade J Sun Z Pagliano O Guillaume P Luescher IF Sander C. CD8(+) T cells specific for tumor antigens can be rendered dysfunctional by the tumor microenvironment through upregulation of the inhibitory receptors BTLA and PD-1. Cancer Res (2012) 72(4):887–96. doi: 10.1158/0008-5472.CAN-11-2637 PMC3288235 22205715

[121] Chapoval AI Ni J Lau JS Wilcox RA Flies DB Liu D. B7-H3: a costimulatory molecule for T cell activation and IFN-gamma production. Nat Immunol (2001) 2(3):269–74. doi: 10.1038/85339 11224528

[122] Castellanos JR Purvis IJ Labak CM Guda MR Tsung AJ Velpula KK. B7-H3 role in the immune landscape of cancer. Am J Clin Exp Immunol (2017) 6(4):66–75. 28695059 PMC5498853

[123] Sun M Richards S Prasad DV Mai XM Rudensky A Dong C. Characterization of mouse and human B7-H3 genes. J Immunol (2002) 168(12):6294–7. doi: 10.4049/jimmunol.168.12.6294 12055244

[124] Steinberger P Majdic O Derdak SV Pfistershammer K Kirchberger S Klauser C. Molecular characterization of human 4Ig-B7-H3, a member of the B7 family with four ig-like domains. J Immunol (2004) 172(4):2352–9. doi: 10.4049/jimmunol.172.4.2352 14764704

[125] Picarda E Ohaegbulam KC Zang X. Molecular pathways: Targeting B7-H3 (CD276) for human cancer immunotherapy. Clin Cancer Res (2016) 22(14):3425–31. doi: 10.1158/1078-0432.CCR-15-2428 PMC4947428 27208063

[126] Suh WK Gajewska BU Okada H Gronski MA Bertram EM Dawicki W. The B7 family member B7-H3 preferentially down-regulates T helper type 1-mediated immune responses. Nat Immunol (2003) 4(9):899–906. doi: 10.1038/ni967 12925852

[127] Hofmeyer KA Ray A Zang X. The contrasting role of B7-H3. Proc Natl Acad Sci U.S.A. (2008) 105(30):10277–8. doi: 10.1073/pnas.0805458105 PMC2492485 18650376

[128] Mahnke K Ring S Johnson TS Schallenberg S Schonfeld K Storn V. Induction of immunosuppressive functions of dendritic cells *in vivo* by CD4+CD25+ regulatory T cells: role of B7-H3 expression and antigen presentation. Eur J Immunol (2007) 37(8):2117–26. doi: 10.1002/eji.200636841 17615586

[129] Han S Wang Y Shi X Zong L Liu L Zhang J. Negative roles of B7-H3 and B7-H4 in the microenvironment of cervical cancer. Exp Cell Res (2018) 371(1):222–30. doi: 10.1016/j.yexcr.2018.08.014 30099052

[130] Lim S Liu H Madeira da Silva L Arora R Liu Z Phillips JB. Immunoregulatory protein B7-H3 reprograms glucose metabolism in cancer cells by ROS-mediated stabilization of HIF1alpha. Cancer Res (2016) 76(8):2231–42. doi: 10.1158/0008-5472.CAN-15-1538 PMC4874665 27197253

[131] Zang X Loke P Kim J Murphy K Waitz R Allison JP. B7x: a widely expressed B7 family member that inhibits T cell activation. Proc Natl Acad Sci U.S.A. (2003) 100(18):10388–92. doi: 10.1073/pnas.1434299100 PMC193571 12920180

[132] Tringler B Zhuo S Pilkington G Torkko KC Singh M Lucia MS. B7-h4 is highly expressed in ductal and lobular breast cancer. Clin Cancer Res (2005) 11(5):1842–8. doi: 10.1158/1078-0432.CCR-04-1658 15756008

[133] Mao YX Chen YJ Ge Y Ma HB Yu JF Wu HY. Recombinant human B7-H4 expressed in escherichia coli inhibits T lymphocyte proliferation and IL-2 secretion *in vitro* . Acta Pharmacol Sin (2006) 27(6):741–6. doi: 10.1111/j.1745-7254.2006.00338.x 16723094

[134] Wang X Hao J Metzger DL Ao Z Chen L Ou D. JNK, p38, and AKT activation. PloS One (2012) 7(1):e28232. doi: 10.1371/journal.pone.0028232 22238573 PMC3251556

[135] Podojil JR Liu LN Marshall SA Chiang MY Goings GE Chen L. B7-H4Ig inhibits mouse and human T-cell function and treats EAE *via* IL-10/Treg-dependent mechanisms. J Autoimmun (2013) 44:71–81. doi: 10.1016/j.jaut.2013.04.001 23683881 PMC3973032

[136] Wei J Loke P Zang X Allison JP. Tissue-specific expression of B7x protects from CD4 T cell-mediated autoimmunity. J Exp Med (2011) 208(8):1683–94. doi: 10.1084/jem.20100639 PMC3149222 21727190

[137] Shen L Qian Y Wu W Weng T Wang FXC Hong B. B7-H4 is a prognostic biomarker for poor survival in patients with pancreatic cancer. Hum Pathol (2017) 66:79–85. doi: 10.1016/j.humpath.2017.05.023 28600225

[138] Krambeck AE Thompson RH Dong H Lohse CM Park ES Kuntz SM. B7-H4 expression in renal cell carcinoma and tumor vasculature: associations with cancer progression and survival. Proc Natl Acad Sci U.S.A. (2006) 103(27):10391–6. doi: 10.1073/pnas.0600937103 PMC1502468 16798883

[139] Podojil JR Miller SD. Potential targeting of B7-H4 for the treatment of cancer. Immunol Rev (2017) 276(1):40–51. doi: 10.1111/imr.12530 28258701 PMC5630270

[140] Smith JB Stashwick C Powell DJ. B7-H4 as a potential target for immunotherapy for gynecologic cancers: a closer look. Gynecol Oncol (2014) 134(1):181–9. doi: 10.1016/j.ygyno.2014.03.553 PMC4066406 24657487

[141] Zhu Y Yao S Iliopoulou BP Han X Augustine MM Xu H. B7-H5 costimulates human T cells *via* CD28H. Nat Commun (2013) 4:2043. doi: 10.1038/ncomms3043 23784006 PMC3698612

[142] Ni L Dong C. New B7 family checkpoints in human cancers. Mol Cancer Ther (2017) 16(7):1203–11. doi: 10.1158/1535-7163.MCT-16-0761 PMC5568666 28679835

[143] Janakiram M Chinai JM Zhao A Sparano JA Zang X. HHLA2 and TMIGD2: new immunotherapeutic targets of the B7 and CD28 families. Oncoimmunology (2015) 4(8):e1026534. doi: 10.1080/2162402X.2015.1026534 26405587 PMC4570140

[144] Rieder SA Wang J White N Qadri A Menard C Stephens G. B7-H7 (HHLA2) inhibits T-cell activation and proliferation in the presence of TCR and CD28 signaling. Cell Mol Immunol (2021) 18(6):1503–11. doi: 10.1038/s41423-020-0361-7 PMC8166953 32005952

[145] Bhatt RS Berjis A Konge JC Mahoney KM Klee AN Freeman SS. KIR3DL3 is an inhibitory receptor for HHLA2 that mediates an alternative immunoinhibitory pathway to PD1. Cancer Immunol Res (2021) 9(2):156–69. doi: 10.1158/2326-6066.CIR-20-0315 PMC8284010 33229411

[146] Zhao R Chinai JM Buhl S Scandiuzzi L Ray A Jeon H. HHLA2 is a member of the B7 family and inhibits human CD4 and CD8 T-cell function. Proc Natl Acad Sci U.S.A. (2013) 110(24):9879–84. doi: 10.1073/pnas.1303524110 PMC3683785 23716685

[147] Qi Y Deng G Xu P Zhang H Yuan F Geng R. HHLA2 is a novel prognostic predictor and potential therapeutic target in malignant glioma. Oncol Rep (2019) 42(6):2309–22. doi: 10.3892/or.2019.7343 PMC6826309 31578594

[148] Zhou QH Li KW Chen X He HX Peng SM Peng SR. HHLA2 and PD-L1 co-expression predicts poor prognosis in patients with clear cell renal cell carcinoma. J Immunother Cancer (2020) 8(1):e000157. doi: 10.1136/jitc-2019-000157 31959726 PMC7057441

[149] Lines JL Pantazi E Mak J Sempere LF Wang L O'Connell S. VISTA is an immune checkpoint molecule for human T cells. Cancer Res (2014) 74(7):1924–32. doi: 10.1158/0008-5472.CAN-13-1504 PMC3979527 24691993

[150] Wang L Rubinstein R Lines JL Wasiuk A Ahonen C Guo Y. VISTA, a novel mouse ig superfamily ligand that negatively regulates T cell responses. J Exp Med (2011) 208(3):577–92. doi: 10.1084/jem.20100619 PMC3058578 21383057

[151] Wang L Le Mercier I Putra J Chen W Liu J Schenk AD. Disruption of the immune-checkpoint VISTA gene imparts a proinflammatory phenotype with predisposition to the development of autoimmunity. Proc Natl Acad Sci U.S.A. (2014) 111(41):14846–51. doi: 10.1073/pnas.1407447111 PMC4205642 25267631

[152] ElTanbouly MA Schaafsma E Smits NC Shah P Cheng C Burns C. VISTA re-programs macrophage biology through the combined regulation of tolerance and anti-inflammatory pathways. Front Immunol (2020) 11:580187. doi: 10.3389/fimmu.2020.580187 33178206 PMC7593571

[153] Han X Vesely MD Yang W Sanmamed MF Badri T Alawa J. PD-1H (VISTA)-mediated suppression of autoimmunity in systemic and cutaneous lupus erythematosus. Sci Transl Med (2019) 11(522):eaax1159. doi: 10.1126/scitranslmed.aax1159 31826980

[154] Le Mercier I Chen W Lines JL Day M Li J Sergent P. VISTA regulates the development of protective antitumor immunity. Cancer Res (2014) 74(7):1933–44. doi: 10.1158/0008-5472.CAN-13-1506 PMC4116689 24691994

[155] Xu W Dong J Zheng Y Zhou J Yuan Y Ta HM. Immune-checkpoint protein VISTA regulates antitumor immunity by controlling myeloid cell-mediated inflammation and immunosuppression. Cancer Immunol Res (2019) 7(9):1497–510. doi: 10.1158/2326-6066.CIR-18-0489 PMC6726548 31340983

[156] Zhai L Spranger S Binder DC Gritsina G Lauing KL Giles FJ. Molecular pathways: Targeting IDO1 and other tryptophan dioxygenases for cancer immunotherapy. Clin Cancer Res (2015) 21(24):5427–33. doi: 10.1158/1078-0432.CCR-15-0420 PMC4681601 26519060

[157] Amobi A Qian F Lugade AA Odunsi K. Tryptophan catabolism and cancer immunotherapy targeting IDO mediated immune suppression. Adv Exp Med Biol (2017) 1036:129–44. doi: 10.1007/978-3-319-67577-0_9 29275469

[158] Prendergast GC Malachowski WJ Mondal A Scherle P Muller AJ. Indoleamine 2,3-dioxygenase and its therapeutic inhibition in cancer. Int Rev Cell Mol Biol (2018) 336:175–203. doi: 10.1016/bs.ircmb.2017.07.004 29413890 PMC6054468

[159] Ufermann CM Domrose A Babel T Tersteegen A Cengiz SC Eller SK. Indoleamine 2,3-dioxygenase activity during acute toxoplasmosis and the suppressed T cell proliferation in mice. Front Cell Infect Microbiol (2019) 9:184. doi: 10.3389/fcimb.2019.00184 31231617 PMC6561234

[160] Mellor A. Indoleamine 2,3 dioxygenase and regulation of T cell immunity. Biochem Biophys Res Commun (2005) 338(1):20–4. doi: 10.1016/j.bbrc.2005.08.232 16157293

[161] Munn DH Mellor AL. IDO in the tumor microenvironment: Inflammation, counter-regulation, and tolerance. Trends Immunol (2016) 37(3):193–207. doi: 10.1016/j.it.2016.01.002 26839260 PMC4916957

[162] Yang Y Liu K Chen Y Gong Y Liang Y. Indoleamine 2,3-dioxygenase (IDO) regulates Th17/Treg immunity in experimental IgA nephropathy. Folia Biol (Praha) (2019) 65(2):101–8. 10.14712/fb2019065020101 31464185

[163] Azadegan-Dehkordi F Shirzad H Ahmadi R Bashash D Abdollahpour-Alitappeh M Luzza F. Increased indoleamine 2, 3-dioxygenase expression modulates Th1/Th17/Th22 and treg pathway in humans with helicobacter pylori-infected gastric mucosa. Hum Immunol (2021) 82(1):46–53. doi: 10.1016/j.humimm.2020.10.005 33127161 PMC8414194

[164] Wang XF Wang HS Wang H Zhang F Wang KF Guo Q. The role of indoleamine 2,3-dioxygenase (IDO) in immune tolerance: focus on macrophage polarization of THP-1 cells. Cell Immunol (2014) 289(1-2):42–8. doi: 10.1016/j.cellimm.2014.02.005 24721110

[165] Jiang N Zhang L Zhao G Lin J Wang Q Xu Q. Indoleamine 2,3-dioxygenase regulates macrophage recruitment, polarization and phagocytosis in aspergillus fumigatus keratitis. Invest Ophthalmol Vis Sci (2020) 61(8):28. doi: 10.1167/iovs.61.8.28 PMC7425693 32692841

[166] Schramme F Crosignani S Frederix K Hoffmann D Pilotte L Stroobant V. Inhibition of tryptophan-dioxygenase activity increases the antitumor efficacy of immune checkpoint inhibitors. Cancer Immunol Res (2020) 8(1):32–45. doi: 10.1158/2326-6066.CIR-19-0041 31806638

[167] Greene LI Bruno TC Christenson JL D'Alessandro A Culp-Hill R Torkko K. A role for tryptophan-2,3-dioxygenase in CD8 T-cell suppression and evidence of tryptophan catabolism in breast cancer patient plasma. Mol Cancer Res (2019) 17(1):131–9. doi: 10.1158/1541-7786.MCR-18-0362 PMC6318037 30143553

[168] Hjortso MD Larsen SK Kongsted P Met O Frosig TM Andersen GH. Tryptophan 2,3-dioxygenase (TDO)-reactive T cells differ in their functional characteristics in health and cancer. Oncoimmunology (2015) 4(1):e968480. doi: 10.4161/21624011.2014.968480 25949861 PMC4368150

[169] Hutloff A Dittrich AM Beier KC Eljaschewitsch B Kraft R Anagnostopoulos I. ICOS is an inducible T-cell co-stimulator structurally and functionally related to CD28. Nature (1999) 397(6716):263–6. doi: 10.1038/16717 9930702

[170] Greenwald RJ Freeman GJ Sharpe AH. The B7 family revisited. Annu Rev Immunol (2005) 23:515–48. doi: 10.1146/annurev.immunol.23.021704.115611 15771580

[171] Witsch EJ Peiser M Hutloff A Buchner K Dorner BG Jonuleit H. ICOS and CD28 reversely regulate IL-10 on re-activation of human effector T cells with mature dendritic cells. Eur J Immunol (2002) 32(9):2680–6. doi: 10.1002/1521-4141(200209)32:9<2680::AID-IMMU2680>3.0.CO;2-6 12207353

[172] Gonzalo JA Tian J Delaney T Corcoran J Rottman JB Lora J. ICOS is critical for T helper cell-mediated lung mucosal inflammatory responses. Nat Immunol (2001) 2(7):597–604. doi: 10.1038/89739 11429543

[173] Coyle AJ Lehar S Lloyd C Tian J Delaney T Manning S. The CD28-related molecule ICOS is required for effective T cell-dependent immune responses. Immunity (2000) 13(1):95–105. doi: 10.1016/s1074-7613(00)00011-x 10933398

[174] Panneton V Chang J Witalis M Li J Suh WK. Inducible T-cell co-stimulator: Signaling mechanisms in T follicular helper cells and beyond. Immunol Rev (2019) 291(1):91–103. doi: 10.1111/imr.12771 31402504

[175] Amatore F Gorvel L Olive D. Role of inducible Co-stimulator (ICOS) in cancer immunotherapy. Expert Opin Biol Ther (2020) 20(2):141–50. doi: 10.1080/14712598.2020.1693540 31738626

[176] Specenier P. Ipilimumab in melanoma. Expert Rev Anticancer Ther (2016) 16(8):811–26. doi: 10.1080/14737140.2016.1211936 27403706

[177] Hodi FS O'Day SJ McDermott DF Weber RW Sosman JA Haanen JB. Improved survival with ipilimumab in patients with metastatic melanoma. N Engl J Med (2010) 363(8):711–23. doi: 10.1056/NEJMoa1003466 PMC3549297 20525992

[178] Eggermont AM Chiarion-Sileni V Grob JJ Dummer R Wolchok JD Schmidt H. Adjuvant ipilimumab versus placebo after complete resection of high-risk stage III melanoma (EORTC 18071): a randomised, double-blind, phase 3 trial. Lancet Oncol (2015) 16(5):522–30. doi: 10.1016/S1470-2045(15)70122-1 25840693

[179] Ascierto PA Del Vecchio M Robert C Mackiewicz A Chiarion-Sileni V Arance A. Ipilimumab 10 mg/kg versus ipilimumab 3 mg/kg in patients with unresectable or metastatic melanoma: a randomised, double-blind, multicentre, phase 3 trial. Lancet Oncol (2017) 18(5):611–22. doi: 10.1016/S1470-2045(17)30231-0 28359784

[180] Hodi FS Butler M Oble DA Seiden MV Haluska FG Kruse A. Immunologic and clinical effects of antibody blockade of cytotoxic T lymphocyte-associated antigen 4 in previously vaccinated cancer patients. Proc Natl Acad Sci U.S.A. (2008) 105(8):3005–10. doi: 10.1073/pnas.0712237105 PMC2268575 18287062

[181] Hodi FS Mihm MC Soiffer RJ Haluska FG Butler M Seiden MV. Biologic activity of cytotoxic T lymphocyte-associated antigen 4 antibody blockade in previously vaccinated metastatic melanoma and ovarian carcinoma patients. Proc Natl Acad Sci U.S.A. (2003) 100(8):4712–7. doi: 10.1073/pnas.0830997100 PMC153621 12682289

[182] Small EJ Tchekmedyian NS Rini BI Fong L Lowy I Allison JP. A pilot trial of CTLA-4 blockade with human anti-CTLA-4 in patients with hormone-refractory prostate cancer. Clin Cancer Res (2007) 13(6):1810–5. doi: 10.1158/1078-0432.CCR-06-2318 17363537

[183] Robert C Thomas L Bondarenko I O'Day S Weber J Garbe C. Ipilimumab plus dacarbazine for previously untreated metastatic melanoma. N Engl J Med (2011) 364(26):2517–26. doi: 10.1056/NEJMoa1104621 21639810

[184] Ribas A Hanson DC Noe DA Millham R Guyot DJ Bernstein SH. Tremelimumab (CP-675,206), a cytotoxic T lymphocyte associated antigen 4 blocking monoclonal antibody in clinical development for patients with cancer. Oncologist (2007) 12(7):873–83. doi: 10.1634/theoncologist.12-7-873 17673618

[185] Tarhini AA. Tremelimumab: a review of development to date in solid tumors. Immunotherapy (2013) 5(3):215–29. doi: 10.2217/imt.13.9 23444951

[186] Ribas A Camacho LH Lopez-Berestein G Pavlov D Bulanhagui CA Millham R. Antitumor activity in melanoma and anti-self responses in a phase I trial with the anti-cytotoxic T lymphocyte-associated antigen 4 monoclonal antibody CP-675,206. J Clin Oncol (2005) 23(35):8968–77. doi: 10.1200/JCO.2005.01.109 16204013

[187] Camacho LH Antonia S Sosman J Kirkwood JM Gajewski TF Redman B. Phase I/II trial of tremelimumab in patients with metastatic melanoma. J Clin Oncol (2009) 27(7):1075–81. doi: 10.1200/JCO.2008.19.2435 19139427

[188] Kirkwood JM Lorigan P Hersey P Hauschild A Robert C McDermott D. Phase II trial of tremelimumab (CP-675,206) in patients with advanced refractory or relapsed melanoma. Clin Cancer Res (2010) 16(3):1042–8. doi: 10.1158/1078-0432.CCR-09-2033 20086001

[189] Ribas A Hauschild A Kefford R Punt CJ Haanen JB Marmol M. Open-label, randomized, comparative study of tremelimumab (CP-675,206) and chemotherapy (temozolomide [TMZ] or dacarbazine [DTIC]) in patients with advanced melanoma. J Clin Oncol (2008) 26(15_suppl):LBA9011–LBA9011. doi: 10.1200/jco.2008.26.15_suppl.lba9011

[190] Ralph C Elkord E Burt DJ O'Dwyer JF Austin EB Stern PL. Modulation of lymphocyte regulation for cancer therapy: a phase II trial of tremelimumab in advanced gastric and esophageal adenocarcinoma. Clin Cancer Res (2010) 16(5):1662–72. doi: 10.1158/1078-0432.CCR-09-2870 20179239

[191] Zatloukal P Heo DS Park K Kang J Butts C Bradford D. Randomized phase II clinical trial comparing tremelimumab (CP-675,206) with best supportive care (BSC) following first-line platinum-based therapy in patients (pts) with advanced non-small cell lung cancer (NSCLC). J Clin Oncol (2009) 27(15_suppl):8071–1. doi: 10.1200/jco.2009.27.15_suppl.8071

[192] Melero I Sangro B Riezu-Boj JI Inãarrairaegui M Lasarte JJ Sarobe P. Prieto: Abstract 4387: Antiviral and antitumoral effects of the anti-CTLA4 agent tremelimumab in patients with hepatocellular carcinoma (HCC) and chronic hepatitis c virus (HCV) infection: Results from a phase II clinical trial. Cancer Res (2012) 72(8_Supplement):4387–7. doi: 10.1158/1538-7445.Am2012-4387

[193] Chung KY Gore I Fong L Venook A Beck SB Dorazio P. Phase II study of the anti-cytotoxic T-lymphocyte-associated antigen 4 monoclonal antibody, tremelimumab, in patients with refractory metastatic colorectal cancer. J Clin Oncol (2010) 28(21):3485–90. doi: 10.1200/JCO.2010.28.3994 20498386

[194] Ribas A Wolchok JD. Cancer immunotherapy using checkpoint blockade. Science (2018) 359(6382):1350–5. doi: 10.1126/science.aar4060 PMC7391259 29567705

[195] Korman AJ Garrett-Thomson SC Lonberg N. The foundations of immune checkpoint blockade and the ipilimumab approval decennial. Nat Rev Drug Discov (2021) 21(7):509–28. doi: 10.1038/s41573-021-00345-8 34937915

[196] Brahmer JR Drake CG Wollner I Powderly JD Picus J Sharfman WH. Phase I study of single-agent anti-programmed death-1 (MDX-1106) in refractory solid tumors: safety, clinical activity, pharmacodynamics, and immunologic correlates. J Clin Oncol (2010) 28(19):3167–75. doi: 10.1200/JCO.2009.26.7609 PMC4834717 20516446

[197] Topalian SL Hodi FS Brahmer JR Gettinger SN Smith DC McDermott DF. Safety, activity, and immune correlates of anti-PD-1 antibody in cancer. N Engl J Med (2012) 366(26):2443–54. doi: 10.1056/NEJMoa1200690 PMC3544539 22658127

[198] Weber JS D'Angelo SP Minor D Hodi FS Gutzmer R Neyns B. Nivolumab versus chemotherapy in patients with advanced melanoma who progressed after anti-CTLA-4 treatment (CheckMate 037): a randomised, controlled, open-label, phase 3 trial. Lancet Oncol (2015) 16(4):375–84. doi: 10.1016/S1470-2045(15)70076-8 25795410

[199] Motzer RJ Escudier B McDermott DF George S Hammers HJ Srinivas S. Nivolumab versus everolimus in advanced renal-cell carcinoma. N Engl J Med (2015) 373(19):1803–13. doi: 10.1056/NEJMoa1510665 PMC5719487 26406148

[200] Borghaei H Paz-Ares L Horn L Spigel DR Steins M Ready NE. Nivolumab versus docetaxel in advanced nonsquamous non-Small-Cell lung cancer. N Engl J Med (2015) 373(17):1627–39. doi: 10.1056/NEJMoa1507643 PMC5705936 26412456

[201] Brahmer J Reckamp KL Baas P Crino L Eberhardt WE Poddubskaya E. Nivolumab versus docetaxel in advanced squamous-cell non-Small-Cell lung cancer. N Engl J Med (2015) 373(2):123–35. doi: 10.1056/NEJMoa1504627 PMC4681400 26028407

[202] Vaddepally RK Kharel P Pandey R Garje R Chandra AB. Review of indications of FDA-approved immune checkpoint inhibitors per NCCN guidelines with the level of evidence. Cancers (Basel) (2020) 12(3):738. doi: 10.3390/cancers12030738 PMC7140028 32245016

[203] Green MR Monti S Rodig SJ Juszczynski P Currie T O'Donnell E. Integrative analysis reveals selective 9p24.1 amplification, increased PD-1 ligand expression, and further induction *via* JAK2 in nodular sclerosing Hodgkin lymphoma and primary mediastinal large b-cell lymphoma. Blood (2010) 116(17):3268–77. doi: 10.1182/blood-2010-05-282780 PMC2995356 20628145

[204] Roemer MG Advani RH Ligon AH Natkunam Y Redd RA Homer H. PD-L1 and PD-L2 genetic alterations define classical Hodgkin lymphoma and predict outcome. J Clin Oncol (2016) 34(23):2690–7. doi: 10.1200/JCO.2016.66.4482 PMC5019753 27069084

[205] Ansell SM Lesokhin AM Borrello I Halwani A Scott EC Gutierrez M. PD-1 blockade with nivolumab in relapsed or refractory hodgkin's lymphoma. N Engl J Med (2015) 372(4):311–9. doi: 10.1056/NEJMoa1411087 PMC4348009 25482239

[206] Armand P Engert A Younes A Fanale M Santoro A Zinzani PL. Nivolumab for Relapsed/Refractory classic Hodgkin lymphoma after failure of autologous hematopoietic cell transplantation: Extended follow-up of the multicohort single-arm phase II CheckMate 205 trial. J Clin Oncol (2018) 36(14):1428–39. doi: 10.1200/JCO.2017.76.0793 PMC6075855 29584546

[207] Kasamon YL de Claro RA Wang Y Shen YL Farrell AT Pazdur R. FDA Approval summary: Nivolumab for the treatment of relapsed or progressive classical Hodgkin lymphoma. Oncologist (2017) 22(5):585–91. doi: 10.1634/theoncologist.2017-0004 PMC5423515 28438889

[208] van Vugt MJH Stone JA De Greef R Snyder ES Lipka L Turner DC. Immunogenicity of pembrolizumab in patients with advanced tumors. J Immunother Cancer (2019) 7(1):212. doi: 10.1186/s40425-019-0663-4 31395089 PMC6686242

[209] Robert C Ribas A Wolchok JD Hodi FS Hamid O Kefford R. Anti-programmed-death-receptor-1 treatment with pembrolizumab in ipilimumab-refractory advanced melanoma: a randomised dose-comparison cohort of a phase 1 trial. Lancet (2014) 384(9948):1109–17. doi: 10.1016/S0140-6736(14)60958-2 25034862

[210] Robert C Schachter J Long GV Arance A Grob JJ Mortier L. Pembrolizumab versus ipilimumab in advanced melanoma. N Engl J Med (2015) 372(26):2521–32. doi: 10.1056/NEJMoa1503093 25891173

[211] Reck M Rodriguez-Abreu D Robinson AG Hui R Csoszi T Fulop A. Pembrolizumab versus chemotherapy for PD-L1-Positive non-Small-Cell lung cancer. N Engl J Med (2016) 375(19):1823–33. doi: 10.1056/NEJMoa1606774 27718847

[212] Shalhout SZ Emerick KS Kaufman HL Miller DM. Immunotherapy for non-melanoma skin cancer. Curr Oncol Rep (2021) 23(11):125. doi: 10.1007/s11912-021-01120-z 34448958 PMC8395379

[213] Akinboro O Larkins E Pai-Scherf LH Mathieu LN Ren Y Cheng J. FDA Approval summary: Pembrolizumab, atezolizumab, and cemiplimab-rwlc as single agents for first-line treatment of advanced/metastatic PD-L1 high NSCLC. Clin Cancer Res (2022) 28(11):2221–8. doi: 10.1158/1078-0432.CCR-21-3844 35101885

[214] Keeping S Xu Y Chen CI Cope S Mojebi A Kuznik A. Comparative efficacy of cemiplimab versus other systemic treatments for advanced cutaneous squamous cell carcinoma. Future Oncol (2021) 17(5):611–27. doi: 10.2217/fon-2020-0823 33052055

[215] Gambale E Fancelli S Caliman E Petrella MC Doni L Pillozzi S. Immune checkpoint blockade with anti-programmed cell death 1 (PD-1) monoclonal antibody (mAb) cemiplimab: ongoing and future perspectives in rare genital cancers treatment. J Immunother Cancer (2022) 10(1):e003540. doi: 10.1136/jitc-2021-003540 35101944 PMC8804682

[216] Yi M Zheng X Niu M Zhu S Ge H Wu K. Combination strategies with PD-1/PD-L1 blockade: current advances and future directions. Mol Cancer (2022) 21(1):28. doi: 10.1186/s12943-021-01489-2 35062949 PMC8780712

[217] Akinleye A Rasool Z. Immune checkpoint inhibitors of PD-L1 as cancer therapeutics. J Hematol Oncol (2019) 12(1):92. doi: 10.1186/s13045-019-0779-5 31488176 PMC6729004

[218] Li M Zhao R Chen J Tian W Xia C Liu X. Next generation of anti-PD-L1 atezolizumab with enhanced anti-tumor efficacy *in vivo* . Sci Rep (2021) 11(1):5774. doi: 10.1038/s41598-021-85329-9 33707569 PMC7952408

[219] Lipson EJ Tawbi HA-H Schadendorf D Ascierto PA Matamala L Gutiérrez EC. Relatlimab (RELA) plus nivolumab (NIVO) versus NIVO in first-line advanced melanoma: Primary phase III results from RELATIVITY-047 (CA224-047). J Clin Oncol (2021) 39(15_suppl):9503–3. doi: 10.1200/JCO.2021.39.15_suppl.9503

[220] Paik J. Nivolumab plus relatlimab: First approval. Drugs (2022) 82(8):925–31. doi: 10.1007/s40265-022-01723-1 35543970

[221] Andre P Denis C Soulas C Bourbon-Caillet C Lopez J Arnoux T. Anti-NKG2A mAb is a checkpoint inhibitor that promotes anti-tumor immunity by unleashing both T and NK cells. Cell (2018) 175(7):1731–43.e13. doi: 10.1016/j.cell.2018.10.014 30503213 PMC6292840

[222] Galot R Le Tourneau C Saada-Bouzid E Daste A Even C Debruyne P. A phase II study of monalizumab in patients with recurrent/metastatic squamous cell carcinoma of the head and neck: The I1 cohort of the EORTC-HNCG-1559 UPSTREAM trial. Eur J Cancer (2021) 158:17–26. doi: 10.1016/j.ejca.2021.09.003 34638090

[223] Caratelli S Arriga R Sconocchia T Ottaviani A Lanzilli G Pastore D. *In vitro* elimination of epidermal growth factor receptor-overexpressing cancer cells by CD32A-chimeric receptor T cells in combination with cetuximab or panitumumab. Int J Cancer (2020) 146(1):236–47. doi: 10.1002/ijc.32663 PMC8711771 31479522

[224] Zhou Q Munger ME Veenstra RG Weigel BJ Hirashima M Munn DH. Coexpression of Tim-3 and PD-1 identifies a CD8+ T-cell exhaustion phenotype in mice with disseminated acute myelogenous leukemia. Blood (2011) 117(17):4501–10. doi: 10.1182/blood-2010-10-310425 PMC3099570 21385853

[225] Tian T Li Z. Targeting Tim-3 in cancer with resistance to PD-1/PD-L1 blockade. Front Oncol (2021) 11:731175. doi: 10.3389/fonc.2021.731175 34631560 PMC8492972

[226] Berry S Giraldo N Nguyen P Green B Xu H Ogurtsova A.33rd annual meeting & pre-conference programs of the society for immunotherapy of cancer (SITC 2018). J Immunother Cancer (2018) 6(Suppl 1):115. doi: 10.1186/s40425-018-0423-x 30400822 PMC6220479

[227] Harding JJ Patnaik A Moreno V Stein M Jankowska AM d. Mendizabal NV. A phase Ia/Ib study of an anti-TIM-3 antibody (LY3321367) monotherapy or in combination with an anti-PD-L1 antibody (LY3300054): Interim safety, efficacy, and pharmacokinetic findings in advanced cancers. J Clin Oncol (2019) 37(8_suppl):12–2. doi: 10.1200/JCO.2019.37.8_suppl.12

[228] Kim JM Chen DS. Immune escape to PD-L1/PD-1 blockade: seven steps to success (or failure). Ann Oncol (2016) 27(8):1492–504. doi: 10.1093/annonc/mdw217 27207108

[229] Rozeman EA Hoefsmit EP Reijers ILM Saw RPM Versluis JM Krijgsman O. Survival and biomarker analyses from the OpACIN-neo and OpACIN neoadjuvant immunotherapy trials in stage III melanoma. Nat Med (2021) 27(2):256–63. doi: 10.1038/s41591-020-01211-7 33558721

[230] Motzer RJ Rini BI McDermott DF Aren Frontera O Hammers HJ Carducci MA. Nivolumab plus ipilimumab versus sunitinib in first-line treatment for advanced renal cell carcinoma: extended follow-up of efficacy and safety results from a randomised, controlled, phase 3 trial. Lancet Oncol (2019) 20(10):1370–85. doi: 10.1016/S1470-2045(19)30413-9 PMC7497870 31427204

[231] Overman MJ Lonardi S Wong KYM Lenz HJ Gelsomino F Aglietta M. Durable clinical benefit with nivolumab plus ipilimumab in DNA mismatch repair-Deficient/Microsatellite instability-high metastatic colorectal cancer. J Clin Oncol (2018) 36(8):773–9. doi: 10.1200/JCO.2017.76.9901 29355075

[232] Yau T Kang YK Kim TY El-Khoueiry AB Santoro A Sangro B. Efficacy and safety of nivolumab plus ipilimumab in patients with advanced hepatocellular carcinoma previously treated with sorafenib: The CheckMate 040 randomized clinical trial. JAMA Oncol (2020) 6(11):e204564. doi: 10.1001/jamaoncol.2020.4564 33001135 PMC7530824

[233] Hellmann MD Paz-Ares L Bernabe Caro R Zurawski B Kim SW Carcereny Costa E. Nivolumab plus ipilimumab in advanced non-Small-Cell lung cancer. N Engl J Med (2019) 381(21):2020–31. doi: 10.1056/NEJMoa1910231 31562796

[234] Paz-Ares L Ciuleanu TE Cobo M Schenker M Zurawski B Menezes J. First-line nivolumab plus ipilimumab combined with two cycles of chemotherapy in patients with non-small-cell lung cancer (CheckMate 9LA): an international, randomised, open-label, phase 3 trial. Lancet Oncol (2021) 22(2):198–211. doi: 10.1016/S1470-2045(20)30641-0 33476593

[235] Scherpereel A Antonia S Bautista Y Grossi F Kowalski D Zalcman G. First-line nivolumab plus ipilimumab versus chemotherapy for the treatment of unresectable malignant pleural mesothelioma: patient-reported outcomes in CheckMate 743. Lung Cancer (2022) 167:8–16. doi: 10.1016/j.lungcan.2022.03.012 35367910

[236] Paz-Ares L Dvorkin M Chen Y Reinmuth N Hotta K Trukhin D. Durvalumab plus platinum-etoposide versus platinum-etoposide in first-line treatment of extensive-stage small-cell lung cancer (CASPIAN): a randomised, controlled, open-label, phase 3 trial. Lancet (2019) 394(10212):1929–39. doi: 10.1016/S0140-6736(19)32222-6 31590988

[237] Goldman JW Dvorkin M Chen Y Reinmuth N Hotta K Trukhin D. Durvalumab, with or without tremelimumab, plus platinum-etoposide versus platinum-etoposide alone in first-line treatment of extensive-stage small-cell lung cancer (CASPIAN): updated results from a randomised, controlled, open-label, phase 3 trial. Lancet Oncol (2021) 22(1):51–65. doi: 10.1016/S1470-2045(20)30539-8 33285097

[238] Gao J Navai N Alhalabi O Siefker-Radtke A Campbell MT Tidwell RS. Neoadjuvant PD-L1 plus CTLA-4 blockade in patients with cisplatin-ineligible operable high-risk urothelial carcinoma. Nat Med (2020) 26(12):1845–51. doi: 10.1038/s41591-020-1086-y PMC9768836 33046869

[239] Powles T van der Heijden MS Castellano D Galsky MD Loriot Y Petrylak DP. Durvalumab alone and durvalumab plus tremelimumab versus chemotherapy in previously untreated patients with unresectable, locally advanced or metastatic urothelial carcinoma (DANUBE): a randomised, open-label, multicentre, phase 3 trial. Lancet Oncol (2020) 21(12):1574–88. doi: 10.1016/S1470-2045(20)30541-6 32971005

[240] Chen EX Jonker DJ Loree JM Kennecke HF Berry SR Couture F. Effect of combined immune checkpoint inhibition vs best supportive care alone in patients with advanced colorectal cancer: The Canadian cancer trials group CO.26 study. JAMA Oncol (2020) 6(6):831–8. doi: 10.1001/jamaoncol.2020.0910 PMC7206536 32379280

[241] Planchard D Reinmuth N Orlov S Fischer JR Sugawara S Mandziuk S. ARCTIC: durvalumab with or without tremelimumab as third-line or later treatment of metastatic non-small-cell lung cancer. Ann Oncol (2020) 31(5):609–18. doi: 10.1016/j.annonc.2020.02.006 32201234

[242] Planchard D Yokoi T McCleod MJ Fischer JR Kim YC Ballas M. A phase III study of durvalumab (MEDI4736) with or without tremelimumab for previously treated patients with advanced NSCLC: Rationale and protocol design of the ARCTIC study. Clin Lung Cancer (2016) 17(3):232–236.e1. doi: 10.1016/j.cllc.2016.03.003 27265743

[243] Kelly RJ Lee J Bang YJ Almhanna K Blum-Murphy M Catenacci DVT. Safety and efficacy of durvalumab and tremelimumab alone or in combination in patients with advanced gastric and gastroesophageal junction adenocarcinoma. Clin Cancer Res (2020) 26(4):846–54. doi: 10.1158/1078-0432.CCR-19-2443 PMC7748730 31676670

[244] Siu LL Even C Mesia R Remenar E Daste A Delord JP. Safety and efficacy of durvalumab with or without tremelimumab in patients with PD-L1-Low/Negative recurrent or metastatic HNSCC: The phase 2 CONDOR randomized clinical trial. JAMA Oncol (2019) 5(2):195–203. doi: 10.1001/jamaoncol.2018.4628 30383184 PMC6439564

[245] Kelley RK Sangro B Harris W Ikeda M Okusaka T Kang YK. Safety, efficacy, and pharmacodynamics of tremelimumab plus durvalumab for patients with unresectable hepatocellular carcinoma: Randomized expansion of a phase I/II study. J Clin Oncol (2021) 39(27):2991–3001. doi: 10.1200/JCO.20.03555 34292792 PMC8445563

[246] Necchi A Giannatempo P Raggi D Mariani L Colecchia M Fare E. An open-label randomized phase 2 study of durvalumab alone or in combination with tremelimumab in patients with advanced germ cell tumors (APACHE): Results from the first planned interim analysis. Eur Urol (2019) 75(1):201–3. doi: 10.1016/j.eururo.2018.09.010 30243800

[247] Calabro L Morra A Giannarelli D Amato G D'Incecco A Covre A. Tremelimumab combined with durvalumab in patients with mesothelioma (NIBIT-MESO-1): an open-label, non-randomised, phase 2 study. Lancet Respir Med (2018) 6(6):451–60. doi: 10.1016/S2213-2600(18)30151-6 29773326

[248] Rizvi NA Cho BC Reinmuth N Lee KH Luft A Ahn MJ. Durvalumab with or without tremelimumab vs standard chemotherapy in first-line treatment of metastatic non-small cell lung cancer: The MYSTIC phase 3 randomized clinical trial. JAMA Oncol (2020) 6(5):661–74. doi: 10.1001/jamaoncol.2020.0237 PMC7146551 32271377

[249] Fuerst ML. Durvalumab + tremelimumab improves survival in advanced liver cancer. Oncol Times (2022) 44(4):29. doi: 10.1097/01.Cot.0000821988.50798.78

[250] Long GV Hodi FS Lipson EJ Schadendorf D Ascierto PA Matamala L. Relatlimab and nivolumab versus nivolumab in previously untreated metastatic or unresectable melanoma: Overall survival and response rates from RELATIVITY-047 (CA224-047). J Clin Oncol (2022) 40(36_suppl):360385–5. doi: 10.1200/JCO.2022.40.36_suppl.360385

[251] Gotwals P Cameron S Cipolletta D Cremasco V Crystal A Hewes B. Prospects for combining targeted and conventional cancer therapy with immunotherapy. Nat Rev Cancer (2017) 17(5):286–301. doi: 10.1038/nrc.2017.17 28338065

[252] Obeid M Tesniere A Ghiringhelli F Fimia GM Apetoh L Perfettini JL. Calreticulin exposure dictates the immunogenicity of cancer cell death. Nat Med (2007) 13(1):54–61. doi: 10.1038/nm1523 17187072

[253] Zhu H Shan Y Ge K Lu J Kong W Jia C. Oxaliplatin induces immunogenic cell death in hepatocellular carcinoma cells and synergizes with immune checkpoint blockade therapy. Cell Oncol (Dordr) (2020) 43(6):1203–14. doi: 10.1007/s13402-020-00552-2 PMC12990673 32797385

[254] Qin S Yi M Jiao D Li A Wu K. Distinct roles of VEGFA and ANGPT2 in lung adenocarcinoma and squamous cell carcinoma. J Cancer (2020) 11(1):153–67. doi: 10.7150/jca.34693 PMC6930396 31892982

[255] Viallard C Larrivee B. Tumor angiogenesis and vascular normalization: alternative therapeutic targets. Angiogenesis (2017) 20(4):409–26. doi: 10.1007/s10456-017-9562-9 28660302

[256] Pan C Liu H Robins E Song W Liu D Li Z. Next-generation immuno-oncology agents: current momentum shifts in cancer immunotherapy. J Hematol Oncol (2020) 13(1):29. doi: 10.1186/s13045-020-00862-w 32245497 PMC7119170

[257] Hsieh JJ Purdue MP Signoretti S Swanton C Albiges L Schmidinger M. Renal cell carcinoma. Nat Rev Dis Primers (2017) 3:17009. doi: 10.1038/nrdp.2017.9 28276433 PMC5936048

[258] Socinski MA Jotte RM Cappuzzo F Orlandi F Stroyakovskiy D Nogami N. Atezolizumab for first-line treatment of metastatic nonsquamous NSCLC. N Engl J Med (2018) 378(24):2288–301. doi: 10.1056/NEJMoa1716948 29863955

[259] Motzer R Alekseev B Rha SY Porta C Eto M Powles T. Lenvatinib plus pembrolizumab or everolimus for advanced renal cell carcinoma. N Engl J Med (2021) 384(14):1289–300. doi: 10.1056/NEJMoa2035716 33616314

[260] Choueiri TK Powles T Burotto M Escudier B Bourlon MT Zurawski B. Nivolumab plus cabozantinib versus sunitinib for advanced renal-cell carcinoma. N Engl J Med (2021) 384(9):829–41. doi: 10.1056/NEJMoa2026982 PMC8436591 33657295

[261] Motzer RJ Robbins PB Powles T Albiges L Haanen JB Larkin J. Avelumab plus axitinib versus sunitinib in advanced renal cell carcinoma: biomarker analysis of the phase 3 JAVELIN renal 101 trial. Nat Med (2020) 26(11):1733–41. doi: 10.1038/s41591-020-1044-8 PMC8493486 32895571

[262] Powles T Plimack ER Soulieres D Waddell T Stus V Gafanov R. Pembrolizumab plus axitinib versus sunitinib monotherapy as first-line treatment of advanced renal cell carcinoma (KEYNOTE-426): extended follow-up from a randomised, open-label, phase 3 trial. Lancet Oncol (2020) 21(12):1563–73. doi: 10.1016/S1470-2045(20)30436-8 33284113

[263] Makker V Rasco D Vogelzang NJ Brose MS Cohn AL Mier J. Lenvatinib plus pembrolizumab in patients with advanced endometrial cancer: an interim analysis of a multicentre, open-label, single-arm, phase 2 trial. Lancet Oncol (2019) 20(5):711–8. doi: 10.1016/S1470-2045(19)30020-8 PMC11686814 30922731

[264] Finn RS Qin S Ikeda M Galle PR Ducreux M Kim TY. Atezolizumab plus bevacizumab in unresectable hepatocellular carcinoma. N Engl J Med (2020) 382(20):1894–905. doi: 10.1056/NEJMoa1915745 32402160

[265] Ramos-Casals M Brahmer JR Callahan MK Flores-Chavez A Keegan N Khamashta MA. Immune-related adverse events of checkpoint inhibitors. Nat Rev Dis Primers (2020) 6(1):38. doi: 10.1038/s41572-020-0160-6 32382051 PMC9728094

[266] Ramos-Casals M Lambotte O Kostine M Calabrese L Suarez-Almazor M Bingham C. THU0628 IMMUNE-RELATED ADVERSE EVENTS INDUCED BY CANCER IMMUNOTHERAPIES. BIG DATA ANALYSIS OF 13,051 CASES (IMMUNOCANCER INTERNATIONAL REGISTRY). Ann Rheum Dis (2019) 78(Suppl 2):607–8. doi: 10.1136/annrheumdis-2019-eular.2707

[267] Postow MA Sidlow R Hellmann MD. Immune-related adverse events associated with immune checkpoint blockade. N Engl J Med (2018) 378(2):158–68. doi: 10.1056/NEJMra1703481 29320654

[268] Yoest JM. Clinical features, predictive correlates, and pathophysiology of immune-related adverse events in immune checkpoint inhibitor treatments in cancer: a short review. Immunotargets Ther (2017) 6:73–82. doi: 10.2147/ITT.S126227 PMC5644546 29067284

[269] Parakh S Cebon J Klein O. Delayed autoimmune toxicity occurring several months after cessation of anti-PD-1 therapy. Oncologist (2018) 23(7):849–51. doi: 10.1634/theoncologist.2017-0531 PMC6058335 29666298

[270] Kanjanapan Y Day D Butler MO Wang L Joshua AM Hogg D. Delayed immune-related adverse events in assessment for dose-limiting toxicity in early phase immunotherapy trials. Eur J Cancer (2019) 107:1–7. doi: 10.1016/j.ejca.2018.10.017 30529898

[271] Weber JS Kahler KC Hauschild A. Management of immune-related adverse events and kinetics of response with ipilimumab. J Clin Oncol (2012) 30(21):2691–7. doi: 10.1200/JCO.2012.41.6750 22614989

[272] Sandigursky S Mor A. Immune-related adverse events in cancer patients treated with immune checkpoint inhibitors. Curr Rheumatol Rep (2018) 20(10):65. doi: 10.1007/s11926-018-0770-0 30191417 PMC6488223

[273] Farolfi A Ridolfi L Guidoboni M Nicoletti SV Piciucchi S Valmorri L. Ipilimumab in advanced melanoma: reports of long-lasting responses. Melanoma Res (2012) 22(3):263–70. doi: 10.1097/CMR.0b013e328353e65c 22516968

[274] Pardoll DM. The blockade of immune checkpoints in cancer immunotherapy. Nat Rev Cancer (2012) 12(4):252–64. doi: 10.1038/nrc3239 PMC4856023 22437870

[275] Fecher LA Agarwala SS Hodi FS Weber JS. Ipilimumab and its toxicities: a multidisciplinary approach. Oncologist (2013) 18(6):733–43. doi: 10.1634/theoncologist.2012-0483 PMC4063401 23774827

[276] Boutros C Tarhini A Routier E Lambotte O Ladurie FL Carbonnel F. Safety profiles of anti-CTLA-4 and anti-PD-1 antibodies alone and in combination. Nat Rev Clin Oncol (2016) 13(8):473–86. doi: 10.1038/nrclinonc.2016.58 27141885

[277] Gupta A De Felice KM Loftus EV Jr. Khanna S. Systematic review: colitis associated with anti-CTLA-4 therapy. Aliment Pharmacol Ther (2015) 42(4):406–17. doi: 10.1111/apt.13281 26079306

[278] Ribas A Puzanov I Dummer R Schadendorf D Hamid O Robert C. Pembrolizumab versus investigator-choice chemotherapy for ipilimumab-refractory melanoma (KEYNOTE-002): a randomised, controlled, phase 2 trial. Lancet Oncol (2015) 16(8):908–18. doi: 10.1016/S1470-2045(15)00083-2 PMC9004487 26115796

[279] Xu C Chen YP Du XJ Liu JQ Huang CL Chen L. Comparative safety of immune checkpoint inhibitors in cancer: systematic review and network meta-analysis. BMJ (2018) 363:k4226. doi: 10.1136/bmj.k4226 30409774 PMC6222274

[280] Wallis N Bulanhagui CA Dorazio PC Healey DI Marshall MA Liang JQ. Safety of tremelimumab (CP-675,206) in patients (pts) with advanced cancer. J Clin Oncol (2008) 26(15_suppl):3040–0. doi: 10.1200/jco.2008.26.15_suppl.3040

[281] Larkin J Chiarion-Sileni V Gonzalez R Grob JJ Cowey CL Lao CD. Combined nivolumab and ipilimumab or monotherapy in untreated melanoma. N Engl J Med (2015) 373(1):23–34. doi: 10.1056/NEJMoa1504030 26027431 PMC5698905

[282] Perez-Ruiz E Minute L Otano I Alvarez M Ochoa MC Belsue V. Prophylactic TNF blockade uncouples efficacy and toxicity in dual CTLA-4 and PD-1 immunotherapy. Nature (2019) 569(7756):428–32. doi: 10.1038/s41586-019-1162-y 31043740

[283] Langer CJ Gadgeel SM Borghaei H Papadimitrakopoulou VA Patnaik A Powell SF. Carboplatin and pemetrexed with or without pembrolizumab for advanced, non-squamous non-small-cell lung cancer: a randomised, phase 2 cohort of the open-label KEYNOTE-021 study. Lancet Oncol (2016) 17(11):1497–508. doi: 10.1016/S1470-2045(16)30498-3 PMC6886237 27745820

[284] Schmid P Adams S Rugo HS Schneeweiss A Barrios CH Iwata H. Atezolizumab and nab-paclitaxel in advanced triple-negative breast cancer. N Engl J Med (2018) 379(22):2108–21. doi: 10.1056/NEJMoa1809615 30345906

[285] Dovedi SJ Elder MJ Yang C Sitnikova SI Irving L Hansen A. Design and efficacy of a monovalent bispecific PD-1/CTLA4 antibody that enhances CTLA4 blockade on PD-1(+) activated T cells. Cancer Discov (2021) 11(5):1100–17. doi: 10.1158/2159-8290.CD-20-1445 33419761

[286] Burton EM Tawbi HA. Bispecific antibodies to PD-1 and CTLA4: Doubling down on T cells to decouple efficacy from toxicity. Cancer Discov (2021) 11(5):1008–10. doi: 10.1158/2159-8290.CD-21-0257 33947716

[287] Schumacher TN Schreiber RD. Neoantigens in cancer immunotherapy. Science (2015) 348(6230):69–74. doi: 10.1126/science.aaa4971 25838375

[288] Riaz N Morris L Havel JJ Makarov V Desrichard A Chan TA. The role of neoantigens in response to immune checkpoint blockade. Int Immunol (2016) 28(8):411–9. doi: 10.1093/intimm/dxw019 PMC4986233 27048318

[289] Schreiber RD Old LJ Smyth MJ. Cancer immunoediting: integrating immunity's roles in cancer suppression and promotion. Science (2011) 331(6024):1565–70. doi: 10.1126/science.1203486 21436444

[290] McGranahan N Furness AJ Rosenthal R Ramskov S Lyngaa R Saini SK. Clonal neoantigens elicit T cell immunoreactivity and sensitivity to immune checkpoint blockade. Science (2016) 351(6280):1463–9. doi: 10.1126/science.aaf1490 PMC4984254 26940869

[291] Le DT Uram JN Wang H Bartlett BR Kemberling H Eyring AD. PD-1 blockade in tumors with mismatch-repair deficiency. N Engl J Med (2015) 372(26):2509–20. doi: 10.1056/NEJMoa1500596 PMC4481136 26028255

[292] Le DT Durham JN Smith KN Wang H Bartlett BR Aulakh LK. Mismatch repair deficiency predicts response of solid tumors to PD-1 blockade. Science (2017) 357(6349):409–13. doi: 10.1126/science.aan6733 PMC5576142 28596308

[293] Hugo W Zaretsky JM Sun L Song C Moreno BH Hu-Lieskovan S. Genomic and transcriptomic features of response to anti-PD-1 therapy in metastatic melanoma. Cell (2016) 165(1):35–44. doi: 10.1016/j.cell.2016.02.065 26997480 PMC4808437

[294] Zaretsky JM Garcia-Diaz A Shin DS Escuin-Ordinas H Hugo W Hu-Lieskovan S. Mutations associated with acquired resistance to PD-1 blockade in melanoma. N Engl J Med (2016) 375(9):819–29. doi: 10.1056/NEJMoa1604958 PMC5007206 27433843

[295] Sade-Feldman M Jiao YJ Chen JH Rooney MS Barzily-Rokni M Eliane JP. Resistance to checkpoint blockade therapy through inactivation of antigen presentation. Nat Commun (2017) 8(1):1136. doi: 10.1038/s41467-017-01062-w 29070816 PMC5656607

[296] Miller BC Sen DR Al Abosy R Bi K Virkud YV LaFleur MW. Subsets of exhausted CD8(+) T cells differentially mediate tumor control and respond to checkpoint blockade. Nat Immunol (2019) 20(3):326–36. doi: 10.1038/s41590-019-0312-6 PMC6673650 30778252

[297] Martin AM Nirschl TR Nirschl CJ Francica BJ Kochel CM van Bokhoven A. Paucity of PD-L1 expression in prostate cancer: innate and adaptive immune resistance. Prostate Cancer Prostatic Dis (2015) 18(4):325–32. doi: 10.1038/pcan.2015.39 PMC4641011 26260996

[298] Rudensky AY. Regulatory T cells and Foxp3. Immunol Rev (2011) 241(1):260–8. doi: 10.1111/j.1600-065X.2011.01018.x PMC3077798 21488902

[299] Chaudhary B Elkord E. Regulatory T cells in the tumor microenvironment and cancer progression: Role and therapeutic targeting. Vaccines (Basel) (2016) 4(3):28. doi: 10.3390/vaccines4030028 PMC5041022 27509527

[300] Khaled YS Ammori BJ Elkord E. Myeloid-derived suppressor cells in cancer: recent progress and prospects. Immunol Cell Biol (2013) 91(8):493–502. doi: 10.1038/icb.2013.29 23797066

[301] Xu J Zhang J Wang J. The application of traditional Chinese medicine against the tumor immune escape. J Transl Int Med (2020) 8(4):203–4. doi: 10.2478/jtim-2020-0032 PMC7805287 33511046

[302] Gopalakrishnan V Helmink BA Spencer CN Reuben A Wargo JA. The influence of the gut microbiome on cancer, immunity, and cancer immunotherapy. Cancer Cell (2018) 33(4):570–80. doi: 10.1016/j.ccell.2018.03.015 PMC6529202 29634945

[303] Sautes-Fridman C Petitprez F Calderaro J Fridman WH. Tertiary lymphoid structures in the era of cancer immunotherapy. Nat Rev Cancer (2019) 19(6):307–25. doi: 10.1038/s41568-019-0144-6 31092904

[304] Goc J Germain C Vo-Bourgais TK Lupo A Klein C Knockaert S. Dendritic cells in tumor-associated tertiary lymphoid structures signal a Th1 cytotoxic immune contexture and license the positive prognostic value of infiltrating CD8+ T cells. Cancer Res (2014) 74(3):705–15. doi: 10.1158/0008-5472.CAN-13-1342 24366885

[305] Behr DS Peitsch WK Hametner C Lasitschka F Houben R Schonhaar K. Prognostic value of immune cell infiltration, tertiary lymphoid structures and PD-L1 expression in merkel cell carcinomas. Int J Clin Exp Pathol (2014) 7(11):7610–21. PMC4270630 25550797

[306] Di Caro G Bergomas F Grizzi F Doni A Bianchi P Malesci A. Occurrence of tertiary lymphoid tissue is associated with T-cell infiltration and predicts better prognosis in early-stage colorectal cancers. Clin Cancer Res (2014) 20(8):2147–58. doi: 10.1158/1078-0432.CCR-13-2590 24523438

[307] Vanhersecke L Brunet M Guegan JP Rey C Bougouin A Cousin S. Mature tertiary lymphoid structures predict immune checkpoint inhibitor efficacy in solid tumors independently of PD-L1 expression. Nat Cancer (2021) 2(8):794–802. doi: 10.1038/s43018-021-00232-6 35118423 PMC8809887

[308] Wherry EJ. T Cell exhaustion. Nat Immunol (2011) 12(6):492–9. doi: 10.1038/ni.2035 21739672

[309] Wang X Haswell JR Roberts CW. Molecular pathways: SWI/SNF (BAF) complexes are frequently mutated in cancer–mechanisms and potential therapeutic insights. Clin Cancer Res (2014) 20(1):21–7. doi: 10.1158/1078-0432.CCR-13-0280 PMC3947303 24122795

[310] Gao J Ward JF Pettaway CA Shi LZ Subudhi SK Vence LM. Sharma: VISTA is an inhibitory immune checkpoint that is increased after ipilimumab therapy in patients with prostate cancer. Nat Med (2017) 23(5):551–5. doi: 10.1038/nm.4308 PMC5466900 28346412

[311] Liu D Jenkins RW Sullivan RJ. Mechanisms of resistance to immune checkpoint blockade. Am J Clin Dermatol (2019) 20(1):41–54. doi: 10.1007/s40257-018-0389-y 30259383 PMC6358473

[312] Sullivan RJ Hong DS Tolcher AW Patnaik A Shapiro G Chmielowski B. Initial results from first-in-human study of IPI-549, a tumor macrophage-targeting agent, combined with nivolumab in advanced solid tumors. J Clin Oncol (2018) 36, no. 15_suppl:3013–3. doi: 10.1200/JCO.2018.36.15_SUPPL.3013

[313] Teng MW Ngiow SF Ribas A Smyth MJ. Classifying cancers based on T-cell infiltration and PD-L1. Cancer Res (2015) 75(11):2139–45. doi: 10.1158/0008-5472.CAN-15-0255 PMC4452411 25977340

[314] Dong ZY Wu SP Liao RQ Huang SM Wu YL. Potential biomarker for checkpoint blockade immunotherapy and treatment strategy. Tumour Biol (2016) 37(4):4251–61. doi: 10.1007/s13277-016-4812-9 26779629

[315] Wang Y Hou K Jin Y Bao B Tang S Qi J. Lung adenocarcinoma-specific three-integrin signature contributes to poor outcomes by metastasis and immune escape pathways. J Transl Int Med (2021) 9(4):249–63. doi: 10.2478/jtim-2021-0046 PMC8802404 35136724

[316] Rizvi NA Hellmann MD Snyder A Kvistborg P Makarov V Havel JJ. Cancer immunology. mutational landscape determines sensitivity to PD-1 blockade in non-small cell lung cancer. Science (2015) 348(6230):124–8. doi: 10.1126/science.aaa1348 PMC4993154 25765070

[317] Yarchoan M Hopkins A Jaffee EM. Tumor mutational burden and response rate to PD-1 inhibition. N Engl J Med (2017) 377(25):2500–1. doi: 10.1056/NEJMc1713444 PMC6549688 29262275

