# Supplementary material for: Harnessing the immune system by targeting immune checkpoints: Providing new hope for Oncotherapy
Source: Front Immunol. 2022 Sep 8;13:982026. doi: 10.3389/fimmu.2022.982026 (PMC9498063; doi:10.3389/fimmu.2022.982026)
Supplement: Supplementary file 1 [file Table_1.docx]

**Table 1 FDA approved immune checkpoint inhibitors and their combinational therapies**

| **Regimens** | **Target** | **Indications** | **Approval** | **Dosing** | **Common AEs** | **Ref** |
| --- | --- | --- | --- | --- | --- | --- |
| **Ipilimumab** | **CTLA4** | **Melanoma**, unresectable or metastatic | 2011 | 3mg/kg Q3W X maximum 4 | Fatigue, diarrhea, pruritus, rash, and colitis | (1, 2) |
| **Ipilimumab as adjuvant** |  | **Melanoma** | 2015 | 10mg/kg Q3W X maximum 4, + 10 mg/kg Q12W for up to 3 years or until disease recurrence or unacceptable toxicity |  | (1, 2) |
| **Nivolumab** | **PD-1** | **Melanoma**, unresectable or metastatic | 2014 | 240mg Q2W or 480mg Q4W, until disease progression or unacceptable toxicity | Fatigue, rash, musculoskeletal pain, pruritus, GI symptoms, asthenia, cough, dyspnea, back pain, arthralgia, upper respiratory tract infection, pyrexia, and headache | (2, 3) |
|  |  | **NSCLC**, metastatic, progressive | 2015 |  |  | (2, 3) |
|  |  | **RCC**, advanced, previously treated | 2015 |  |  | (2, 3) |
|  |  | **Hodgkin lymphoma**, classical | 2016 |  |  | (3) |
|  |  | **Head and neck cancer,** squamous cell, recurrent or metastatic | 2016 |  |  | (2, 3) |
|  |  | **Urothelial carcinoma**, locally advanced or metastatic | 2017 |  |  | (2, 3) |
|  |  | **Colorectal cancer**, metastatic, MSI-H or dMMR | 2017 |  |  | (3) |
|  |  | **Esophageal carcinoma**, squamous cell, unresectable advanced, after prior chemotherapy | 2020 |  |  | (3) |
| **Nivolumab as adjuvant** |  | **Melanoma** | 2017 | 240mg Q2W or 480mg Q4W, until disease progression or unacceptable toxicity for up to 1 year |  | (4) |
|  |  | **Esophageal or GEJ cancer**, resected | 2021 |  |  | (5) |
|  |  | **Urothelial carcinoma**, following resection | 2021 |  |  | (6) |
| **Pembrolizumab** | **PD-1** | **Melanoma**, unresectable or metastatic | 2014 | 200mg Q3W or 400mg Q6W, until disease progression or unacceptable toxicity | Fatigue, musculoskeletal pain, GI symptoms, pruritus, rash, pyrexia, cough, dyspnea and pain | (2, 3) |
|  |  | **NSCLC**, advanced | 2015 | 200mg Q3W or 400mg Q6W, until disease progression, unacceptable toxicity or for up to 2 years |  | (2, 3) |
|  |  | **Head and neck cancer,** squamous cell, recurrent or metastatic | 2016 |  |  | (2, 3) |
|  |  | **NSCLC**, metastatic, first-line | 2016 |  |  | (2, 3) |
|  |  | **Hodgkin lymphoma**, classical | 2017 |  |  | (2, 3) |
|  |  | **Urothelial carcinoma**, locally advanced or metastatic | 2017 |  |  | (2, 3) |
|  |  | **MSI-H or dMMR cancer**, unresectable or metastatic | 2017 |  |  | (3, 5) |
|  |  | **Cervical cancer**, recurrent or metastatic, PD-L1 expressing | 2018 |  |  | (2, 5) |
|  |  | **PMBCL**, relapsed or refractory | 2018 |  |  | (2) |
|  |  | **HCC**, previously treated with sorafenib | 2018 |  |  | (2, 5) |
|  |  | **Merkel cell carcinoma**, recurrent or metastatic | 2018 |  |  | (2, 3, 5) |
|  |  | **NSCLC**, stage III, first-line | 2019 |  |  | (3) |
|  |  | **Esophageal carcinoma**, squamous cell, recurrent locally advanced, or metastatic | 2019 |  |  | (3, 5) |
|  |  | **Non-muscle invasive bladder cancer**, BCG-unresponsive, high-risk | 2020 |  |  | (5) |
|  |  | **TMB-H**, unresectable or metastatic, regardless of tumor type | 2020 |  |  | (7, 8) |
|  |  | **cSCC**, recurrent, metastatic | 2020 |  |  | (9) |
|  |  | **Colorectal cancer**, first-line, unresectable or metastatic, MSI-H or dMMR | 2020 |  |  | (10, 11) |
|  |  | **Hodgkin lymphoma**, relapsed or refractory | 2020 |  |  | (3, 5) |
|  |  | **cSCC**, locally advanced | 2021 |  |  | (11) |
|  |  | **Endometrial carcinoma**, MSI-H/dMMR, advanced | 2022 |  |  | (12) |
| **Pembrolizumab as adjuvant** |  | **Melanoma** | 2019 | 200mg Q3W or 400mg Q6W, until disease recurrence, unacceptable toxicity or for up to 1 year |  | (4) |
|  |  | **RCC** | 2021 |  |  | (13) |
|  |  | **Breast cancer**, triple negative, high-risk, early stage | 2021 | 200mg Q3W X 9 or 400mg Q6W X 5, or until disease progression or unacceptable toxicity |  | (14) |
| **Cemiplimab-rwlc** | **PD-1** | **cSCC**, metastatic or locally advanced | 2018 | 350mg Q3W, until disease progression or unacceptable toxicity | Fatigue, rash and  diarrhea | (2, 15) |
|  |  | **Basal cell carcinoma**, locally advanced or metastatic | 2021 |  |  | (15) |
|  |  | **NSCLC**, metastatic or locally advanced, PD-L1 expressing, no EGFR, ALK or ROS1 aberrations, first-line | 2021 |  |  | (15) |
| **Atezolizumab** | **PD-1** | **NSCLC**, metastatic, previously treated, EGFR or ALK gene abnormalities | 2016 | 1200mg Q3W, until disease progression or unacceptable toxicity | Fatigue, asthenia, nausea, cough, dyspnea, and decreased appetite | (4) |
|  |  | **Urothelial carcinoma**, locally advanced or metastatic, cisplatin ineligible | 2017 |  |  | (2) |
|  |  | **NSCLC**, metastatic, PD-L1 expressing, first-line, no EGFR or ALK genomic tumor aberrations | 2020 |  |  | (4) |
| **Atezolizumab as adjuvant** |  | **NSCLC**, stage II-IIIA, following surgery and platinum-based chemotherapy, PD-L1 expressing | 2021 | 1200mg Q3W, until disease recurrence, unacceptable toxicity or for up to 1 year |  | (16) |
| **Avelumab** | **PD-L1** | **Merkel cell carcinoma**, metastatic | 2017 | 800mg Q2W, until disease progression or unacceptable toxicity | Fatigue, musculoskeletal pain, GI symptoms, infusion-related reaction, rash, peripheral edema (in MCC) and urinary tract infection in (in UC) | (2, 3) |
|  |  | **Urothelial carcinoma**, locally advanced or metastatic, progressive disease | 2017 |  |  | (2, 3) |
|  |  | **Urothelial carcinoma**, locally advanced or metastatic, first-line maintenance | 2020 |  |  | (2) |
| **Durvalumab** | **PD-L1** | **NSCLC**, unresectable, stage III | 2018 | 10mg/kg Q2W or 1500mg Q4W, until disease progression or unacceptable toxicity or for up to 1 year | Cough, fatigue, pneumonitis/radiation pneumonitis, upper respiratory tract infections, dyspnea and rash | (2) |
| **Ipilimumab + nivolumab** | **CTLA4/PD-1** | **Melanoma**, unresectable or metastatic | 2015 | (Ipilimumab 3 mg/kg + nivolumab 1 mg/kg) Q3W X maximum 4 or until unacceptable toxicity, + nivolumab 240 mg Q2W or 480 mg Q4W until disease progression or unacceptable toxicity |  | (2) |
|  |  | **RCC**, advanced, intermediate or poor risk, first-line | 2018 | (Ipilimumab 1mg/kg + nivolumab 3mg/kg) Q3W X 4, + nivolumab 240mg Q2W or 480mg Q4W, until disease progression or unacceptable toxicity |  | (2) |
|  |  | **Colorectal cancer**, metastatic, MSI-H or dMMR | 2018 |  |  | (2) |
|  |  | **HCC**, previously  treated with sorafenib | 2020 | (Ipilimumab 3mg/kg + nivolumab 1mg/kg) Q3W X 4, + nivolumab 240mg Q2W or 480mg Q4W until disease progression or unacceptable toxicity |  | (4) |
|  |  | **NSCLC**, metastatic, PD-L1 expressing ≥ 1%, first-line | 2020 | Ipilimumab 1mg/kg Q6W+ nivolumab 3mg/kg Q2W until disease progression or unacceptable toxicity or for up to 2 years |  | (17) |
|  |  | **Malignant Pleural mesothelioma**, unresectable, first-line, | 2020 | Ipilimumab 1mg/kg Q6W + nivolumab 360mg Q3W until disease progression, unacceptable toxicity or for up to 2 years |  | (18) |
|  |  | **Esophageal carcinoma**, squamous cell, unresectable advanced or metastatic, fisrt line | 2022 | Ipilimumab 1mg/kg Q6W+ nivolumab 3mg/kg Q2W or 360mg Q3W until disease progression or unacceptable toxicity or for up to 2 years |  | (19) |
| **Nivolumab + relatlimab** | **PD-1/Lag-3** | **Melanoma**, unresectable or metastatic | 2022 | (Nivolumab 480mg + relatlimab 160mg) Q4W, until disease progression or unacceptable toxicity |  | (20) |
| **Nivolumab + cabozantinib** | **PD-1/VEGF** | **RCC**, advanced, first-line | 2021 | 240mg Q2W or 480mg Q4W + Cabozantinib 40mg qd, until disease progression, unacceptable toxicity or up to 2 years |  | (21) |
| **Pembrolizumab + lenvatinib** | **PD-1/VEGF** | **Endometrial carcinoma**, advanced | 2019 | Pembrolizumab 200mg Q3W or 400mg Q6W + lenvatinib 20mg qd, until disease progression, unacceptable toxicity or for up to 2 years |  | (22) |
|  |  | **RCC**, advanced | 2021 |  |  | (23) |
| **Pembrolizumab + axitinib** | **PD-1/VEGF** | **RCC**, advanced | 2019 | Pembrolizumab 200mg Q3W or 400mg Q6W + axitinib 5mg Q12H (6 weeks later, 2-10mg Q12H based on AEs) until disease progression, unacceptable toxicity or for up to 2 years |  | (24) |
| **Atezolizumab + bevacizumab** | **PD-1/VEGF** | **HCC**, unresectable or metastatic | 2020 | (Atezolizumab 1200mg + bevacizumab 15mg/kg) Q3W, until disease progression or unacceptable toxicity |  | (25) |
| **Atezolizumab + cobimetinib + vemurafenib** |  | **Melanoma**, advanced, BRAF V600 mutation-positive | 2020 | Atezolizumab 840mg Q2W (+ cobimetinib + vemurafenib), until disease progression or unacceptable toxicity |  | (26) |
| **Avelumab + axitinib** | **PD-L1/VEGF** | **RCC**, advanced | 2019 | Avelumab 800mg Q2W + axitinib 5mg Q12H (2 weeks later, 2-10mg Q12H based on AEs), until disease progression or unacceptable toxicity |  | (27) |
| **Nivolumab + chemotherapy** |  | **Gastric cancer, GEJ cancer and Esophageal adenocarcinoma**, advanced or metastatic | 2021 | 240mg Q2W or 360mg Q3W + fluoropyrimidine- and platinum-containing chemotherapy until disease progression, unacceptable toxicity or up to 2 years |  | (28) |
|  |  | **NSCLC,** resectable, neoadjuvant | 2022 | (360mg Q3W + platinum-doublet chemotherapy) X 3 |  | (29) |
|  |  | **Esophageal carcinoma**, squamous cell, unresectable advanced or metastatic, fisrt line | 2022 | 240mg Q2W or 480mg Q4W + fluoropyrimidine- and platinum-containing chemotherapy until disease progression, unacceptable toxicity or up to 2 years |  | (19) |
| **Pembrolizumab + chemotherapy** |  | **NSCLC**, metastatic, nonsquamous, first-line | 2017 | (Pembrolizumab 200mg Q3W or 400mg Q6W + pemetrexed + cisplatin/carboplatin) X 4,+ pembrolizumab 200mg Q3W or 400mg Q6W (± pemetrexed maintenance therapy) until disease progression, unacceptable toxicity or for a total duration of pembrolizumab up to 35 cycles or 2 years |  | (4) |
|  |  | **NSCLC**, metastatic, squamous, first-line | 2018 | (Pembrolizumab 200mg Q3W or 400mg Q6W + carboplatin + paclitaxel) X 4,+ pembrolizumab 200mg Q3W or 400mg Q6W until radiographic disease progression, unacceptable toxicity or for a total duration of pembrolizumab up to 35 cycles |  | (4) |
|  |  | **Head and neck cancer,** squamous cell, unresectable/recurrent or metastatic, first-line | 2019 | Pembrolizumab 200mg Q3W or 400mg Q6W, until disease progression, unacceptable toxicity or up to 2 years, initially + 6 cycles of fluorouracil and either carboplatin or cisplatin |  | (2) |
|  |  | **Breast cancer**, triple negative, locally recurrent unresectable or metastatic, PD-L1 expressing | 2020 | Pembrolizumab 200mg Q3W or 400mg Q6W, until disease progression, unacceptable toxicity or for up to 2 years  Chemotherapy^*^: paclitaxel (protein bound or conventional), or gemcitabine/carboplatin |  | (14) |
|  |  | **Esophageal or GEJ carcinoma**, locally advanced or metastatic | 2021 | Pembrolizumab 200mg Q3W or 400mg Q6W + 6 cycles of fluorouracil and cisplatin, continue pembrolizumab and fluorouracil until disease progression, unacceptable toxicity or for up to 2 years |  | (30) |
|  |  | **Breast cancer**, triple negative, high-risk, early stage | 2021 | (Pembrolizumab 200mg Q3W + paclitaxel and carboplatin) X 4 + (200mg Q3W + cyclophosphamide + doxorubicin/epirubicin) X 4 or 400mg Q6W X 4 or until disease progression or unacceptable toxicity |  | (31) |
|  |  | **Cervical cancer**, persistent, recurrent or metastatic, PD-L1 expressing | 2021 | Pembrolizumab 200mg Q3W or 400mg Q6W, until disease progression, unacceptable toxicity or for up to 2 years  Chemotherapy^*^: paclitaxel + cisplatin/carboplatin  With or without bevacizumab |  | (32) |
| **Atezolizumab + chemotherapy** |  | **SCLC**, extensive stage, first-line | 2019 | (Atezolizumab 1200mg Q3W + carboplatin, etoposide) X 4, + atezolizumab at any approved doses until disease progression or unacceptable toxicity |  | (2) |
|  |  | **NSCLC**, nonsquamous, metastatic, no EGFR or ALK genomic tumor aberrations, first-line | 2019 | (Atezolizumab 1200mg D1 Q3W + paclitaxel [protein-bound], carboplatin) X 4-6, + atezolizumab at any approved doses until disease progression or unacceptable toxicity |  | (4) |
| **Durvalumab + chemotherapy** |  | **SCLC**, extensive-stage | 2020 | (Durvalumab 1500mg Q3W + etoposide + carboplatin/cisplatin) X 4, + durvalumab 1500mg Q4W until disease progression or unacceptable toxicity |  | (33) |
| **Ipilimumab + nivolumab + chemotherapy** |  | **NSCLC**, metastatic or recurrent, first-line | 2020 | Ipilimumab 1 mg/kg Q6W + nivolumab 360mg Q3W + two cycles of histology-based platinum-doublet chemotherapy until disease progression, unacceptable toxicity or for up to 2 years |  | (34) |
| **Pembrolizumab +** **trastuzumab + chemotherapy** |  | **Gastric or GEJ adenocarcinoma**, locally advanced unresectable or metastatic, HER2-positive, first-line | 2021 | Pembrolizumab 200mg Q3W or 400mg Q6W + trastuzumab + fluoropyrimidine- and platinum-containing chemotherapy, until disease progression, unacceptable toxicity or for up to 2 years |  | (35) |
| **Atezolizumab + bevacizumab + chemotherapy** |  | **NSCLC**, nonsquamous, metastatic, no EGFR or ALK genomic tumor aberrations, first-line | 2018 | (Atezolizumab 1200mg D1 Q3W + bevacizumab 15mg/kg Q3W, paclitaxel, carboplatin) X 4-6, + atezolizumab 1200mg D1 (+bevacizumab) Q3W until disease progression or unacceptable toxicity |  | (36) |

Abbreviations: AEs: adverse events; BCG: Bacillus Calmettte-Guerin; cSCC: Cutaneous squamous cell carcinoma; D: day; dMMR: mismatch repair deficient; ESCC: esophageal squamous cell carcinoma; GEJ: gastroesophageal junction; GI: gastrointestinal; HCC: hepatocellular carcinoma; NSCLC: non-small cell lung cancer; MSI-H: microsatellite instability-high; PMBCL: primary mediastinal large B-cell lymphoma; RCC: renal cell carcinoma; SCLC: small cell lung cancer; TMB-H: tumor mutational burden-high.

*: dosage from clinical trials.

**References:**

1. Specenier P. Ipilimumab in melanoma. Expert Rev Anticancer Ther (2016) 16(8):811–26. doi: 10.1080/14737140.2016.1211936

2. Vaddepally RK, Kharel P, Pandey R, Garje R, Chandra AB. Review of indications of FDA-approved immune checkpoint inhibitors per NCCN guidelines

with the level of evidence. Cancers (Basel) (2020) 12(3):738. doi: 10.3390/cancers12030738

3. Ribas A, Wolchok JD. Cancer immunotherapy using checkpoint blockade. Science (2018) 359(6382):1350–5. doi: 10.1126/science.aar4060

4. Bagchi S, Yuan R, Engleman EG. Immune checkpoint inhibitors for the treatment of cancer: Clinical impact and mechanisms of response and resistance.

Annu Rev Pathol (2021) 16:223–49. doi: 10.1146/annurev-pathol-042020-042741

5. Kelly RJ, Ajani JA, Kuzdzal J, Zander T, Van Cutsem E, Piessen G, et al. Adjuvant nivolumab in resected esophageal or gastroesophageal junction cancer. N Engl J Med (2021) 384(13):1191–203. doi: 10.1056/NEJMoa2032125

6. Bajorin DF, Witjes JA, Gschwend JE, Schenker M, Valderrama BP, Tomita Y, et al. Adjuvant nivolumab versus placebo in muscle-invasive urothelial

carcinoma. N Engl J Med (2021) 384(22):2102–14. doi: 10.1056/NEJMoa2034442

7. Cristescu R, Aurora-Garg D, Albright A, Xu L, Liu XQ, Loboda A, et al. Tumor mutational burden predicts the efficacy of pembrolizumab monotherapy: a pan-tumor retrospective analysis of participants with advanced solid tumors. J Immunother Cancer (2022) 10(1):e003091. doi: 10.1136/jitc-2021-003091

8. Marabelle A, Fakih M, Lopez J, Shah M, Shapira-Frommer R, Nakagawa K, et al. Association of tumour mutational burden with outcomes in patients with

advanced solid tumours treated with pembrolizumab: prospective biomarker analysis of the multicohort, open-label, phase 2 KEYNOTE-158 study. Lancet

Oncol (2020) 21(10):1353–65. doi: 10.1016/S1470-2045(20)30445-9

9. United States Food and Drug Administration approves pembrolizumab for cutaneous squamous cell carcinoma. Available at: https://www.fda.gov/drugs/drug-approvals-and-databases/fda-approves-pembrolizumab-cutaneous-squamous-cell-carcinoma.

10. Andre T, Shiu KK, Kim TW, Jensen BV, Jensen LH, Punt C, et al. Pembrolizumab in microsatellite-Instability-High advanced colorectal cancer. N

Engl J Med (2020) 383(23):2207–18. doi: 10.1056/NEJMoa2017699

11. Hughes BGM, Munoz-Couselo E, Mortier L, Bratland A, Gutzmer R, Roshdy O, et al. Pembrolizumab for locally advanced and recurrent/metastatic

cutaneous squamous cell carcinoma (KEYNOTE-629 study): an open-label, nonrandomized, multicenter, phase II trial. Ann Oncol (2021) 32(10):1276–85.

doi: 10.1016/j.annonc.2021.07.008

12. O'Malley DM, Bariani GM, Cassier PA, Marabelle A, Hansen AR, De Jesus Acosta A, et al. Pembrolizumab in patients with microsatellite instability-high

advanced endometrial cancer: Results from the KEYNOTE-158 study. J Clin Oncol (2022) 40(7):752–61. doi: 10.1200/JCO.21.01874

13. Choueiri TK, Tomczak P, Park SH, Venugopal B, Ferguson T, Chang YH, et al. Adjuvant pembrolizumab after nephrectomy in renal-cell carcinoma. N Engl J Med (2021) 385(8):683–94. doi: 10.1056/NEJMoa2106391

14. Cortes J, Cescon DW, Rugo HS, Nowecki Z, Im SA, Yusof MM, et al. Pembrolizumab plus chemotherapy versus placebo plus chemotherapy for

previously untreated locally recurrent inoperable or metastatic triple-negative breast cancer (KEYNOTE-355): a randomised, placebo-controlled, double-blind, phase 3 clinical trial. Lancet (2020) 396(10265):1817–28. doi: 10.1016/S0140-6736(20)32531-9

15. Gambale E, Fancelli S, Caliman E, Petrella MC, Doni L, Pillozzi S, et al. Immune checkpoint blockade with anti-programmed cell death 1 (PD-1)

monoclonal antibody (mAb) cemiplimab: ongoing and future perspectives in rare genital cancers treatment. J Immunother Cancer (2022) 10(1):e003540.

doi: 10.1136/jitc-2021-003540

16. Felip E, Altorki N, Zhou C, Csoszi T, Vynnychenko I, Goloborodko O, et al. Adjuvant atezolizumab after adjuvant chemotherapy in resected stage IB-IIIA

non-small-cell lung cancer (IMpower010): a randomised, multicentre, open-label, phase 3 trial. Lancet (2021) 398(10308):1344–57. doi: 10.1016/S0140-6736(21)02098-5

17. Hellmann MD, Paz-Ares L, Bernabe Caro R, Zurawski B, Kim SW, Carcereny Costa E, et al. Nivolumab plus ipilimumab in advanced non-Small-

Cell lung cancer. N Engl J Med (2019) 381(21):2020–31. doi: 10.1056/NEJMoa1910231

18. Wright K. FDA Approves nivolumab plus ipilimumab for previously untreated unresectable malignant pleural mesothelioma. Oncol (Williston Park)

(2020) 34(11):502–3. doi: 10.46883/ONC.2020.3411.0502

19. Doki Y, Ajani JA, Kato K, Xu J, Wyrwicz L, Motoyama S, et al. Nivolumab combination therapy in advanced esophageal squamous-cell carcinoma. N Engl J Med (2022) 386(5):449–62. doi: 10.1056/NEJMoa2111380

20. Paik J. Nivolumab plus relatlimab: First approval. Drugs (2022) 82(8):925–31. doi: 10.1007/s40265-022-01723-1

21. Choueiri TK, Powles T, Burotto M, Escudier B, Bourlon MT, Zurawski B, et al. Nivolumab plus cabozantinib versus sunitinib for advanced renal-cell

carcinoma. N Engl J Med (2021) 384(9):829–41. doi: 10.1056/NEJMoa2026982

22. Makker V, Colombo N, Casado Herraez A, Santin AD, Colomba E, Miller DS, et al. Lenvatinib plus pembrolizumab for advanced endometrial cancer. N Engl J Med (2022) 386(5):437–48. doi: 10.1056/NEJMoa2108330

23. Motzer R, Alekseev B, Rha SY, Porta C, Eto M, Powles T, et al. Lenvatinib plus pembrolizumab or everolimus for advanced renal cell carcinoma. N Engl J Med (2021) 384(14):1289–300. doi: 10.1056/NEJMoa2035716

24. Rini BI, Plimack ER, Stus V, Gafanov R, Hawkins R, Nosov D, et al. Pembrolizumab plus axitinib versus sunitinib for advanced renal-cell carcinoma. N

Engl J Med (2019) 380(12):1116–27. doi: 10.1056/NEJMoa1816714

25. Finn RS, Qin S, Ikeda M, Galle PR, Ducreux M, Kim TY, et al. Atezolizumab plus bevacizumab in unresectable hepatocellular carcinoma. N

Engl J Med (2020) 382(20):1894–905. doi: 10.1056/NEJMoa1915745

26. Gutzmer R, Stroyakovskiy D, Gogas H, Robert C, Lewis K, Protsenko S, et al. Atezolizumab, vemurafenib, and cobimetinib as first-line treatment for

unresectable advanced BRAF(V600) mutation-positive melanoma (IMspire150): primary analysis of the randomised, double-blind, placebo-controlled, phase 3 trial. Lancet (2020) 395(10240):1835–44. doi: 10.1016/S0140-6736(20)30934-X

27. Motzer RJ, Penkov K, Haanen J, Rini B, Albiges L, Campbell MT, et al. Avelumab plus axitinib versus sunitinib for advanced renal-cell carcinoma. N Engl J Med (2019) 380(12):1103–15. doi: 10.1056/NEJMoa1816047

28. Janjigian YY, Shitara K, Moehler M, Garrido M, Salman P, Shen L, et al. First-line nivolumab plus chemotherapy versus chemotherapy alone for advanced

gastric, gastro-oesophageal junction, and oesophageal adenocarcinoma (CheckMate 649): a randomised, open-label, phase 3 trial. Lancet (2021) 398

(10294):27–40. doi: 10.1016/S0140-6736(21)00797-2

29. Forde PM, Spicer J, Lu S, Provencio M, Mitsudomi T, Awad MM, et al. Neoadjuvant nivolumab plus chemotherapy in resectable lung cancer. N Engl J Med (2022) 386(21):1973–85. doi: 10.1056/NEJMoa2202170

30. Sun JM, Shen L, Shah MA, Enzinger P, Adenis A, Doi T, et al. Pembrolizumab plus chemotherapy versus chemotherapy alone for first-line

treatment of advanced oesophageal cancer (KEYNOTE-590): a randomised, placebo-controlled, phase 3 study. Lancet (2021) 398(10302):759–71.

doi: 10.1016/S0140-6736(21)01234-4

31. Schmid P, Salgado R, Park YH, Munoz-Couselo E, Kim SB, Sohn J, et al. Pembrolizumab plus chemotherapy as neoadjuvant treatment of high-risk, earlystage triple-negative breast cancer: results from the phase 1b open-label, multicohort KEYNOTE-173 study. Ann Oncol (2020) 31(5):569–81.

doi: 10.1016/j.annonc.2020.01.072

32. Colombo N, Dubot C, Lorusso D, Caceres MV, Hasegawa K, Shapira-Frommer R, et al. Pembrolizumab for persistent, recurrent, or metastatic cervical

cancer. N Engl J Med (2021) 385(20):1856–67. doi: 10.1056/NEJMoa2112435

33. Al-Salama ZT. Durvalumab: A review in extensive-stage SCLC. Target Oncol (2021) 16(6):857–64. doi: 10.1007/s11523-021-00843-0

34. Paz-Ares L, Ciuleanu TE, Cobo M, Schenker M, Zurawski B, Menezes J, et al. First-line nivolumab plus ipilimumab combined with two cycles of

chemotherapy in patients with non-small-cell lung cancer (CheckMate 9LA): an international, randomised, open-label, phase 3 trial. Lancet Oncol (2021) 22

(2):198–211. doi: 10.1016/S1470-2045(20)30641-0

35. Chung HC, Bang YJ, S Fuchs C, Qin SK, Satoh T, Shitara K, et al. First-line pembrolizumab/placebo plus trastuzumab and chemotherapy in HER2-positive

advanced gastric cancer: KEYNOTE-811. Future Oncol (2021) 17(5):491–501. doi: 10.2217/fon-2020-0737

36. Socinski MA, Jotte RM, Cappuzzo F, Orlandi F, Stroyakovskiy D, Nogami N, et al. Atezolizumab for first-line treatment of metastatic nonsquamous NSCLC.

N Engl J Med (2018) 378(24):2288–301. doi: 10.1056/NEJMoa1716948
